# Congenital Tumors—Magnetic Resonance Imaging Findings with Focus on Rare Tumors

**DOI:** 10.3390/cancers16010043

**Published:** 2023-12-20

**Authors:** Piotr Kwasniewicz, Julia Wieczorek-Pastusiak, Anna Romaniuk-Doroszewska, Monika Bekiesinska-Figatowska

**Affiliations:** Department of Diagnostic Imaging, Institute of Mother and Child, 01-211 Warsaw, Poland; kwasniewiczp@gmail.com (P.K.); juliawieczorekpastusiak@gmail.com (J.W.-P.); anna.romaniuk.doroszewska@wp.pl (A.R.-D.)

**Keywords:** fetus, infant, congenital tumor, magnetic resonance imaging (MRI)

## Abstract

**Simple Summary:**

Congenital tumors are an uncommon finding on prenatal ultrasound and in the first 3 months of life, and they are (almost) always subjected to magnetic resonance imaging. Although they are usually easy to recognize as pathological masses, differential diagnosis is not easy and includes both benign and malignant conditions. Teratomas are the most frequent group of inborn neoplasms, followed by cardiac rhabdomyomas. In this paper, the authors show a series of cases in order to provide tips to identify the more common masses and to keep in mind that the most unusual tumor may occur as congenital and that no diagnosis should be rejected a priori. The article is intended to raise awareness and draw attention to this little-known group of cancers and facilitate the diagnostic process.

**Abstract:**

Congenital tumors are rare and, owing to this rarity, there is limited information on many of them. A total of 839 fetal and postnatal MRI studies performed in the first 3 months of life were retrospectively reviewed. They were performed with the use of 1.5 T scanners. Seventy-six tumors were diagnosed based on fetal MRI between 20 and 37 gestational weeks, and 27 were found after birth, from 1 day of age to 3 months of life. Teratomas were the most common tumors in our dataset, mainly in the sacrococcygeal region (SCT), followed by cardiac rhabdomyomas and subependymal giant cell astrocytomas (SEGA) associated with TSC, and neuroblastomas. The group of less common tumors consisted of infantile fibrosarcomas, malignant rhabdoid tumors, mesoblastic nephromas and Wilms tumor, craniopharyngiomas, brain stem gliomas, desmoplastic infantile astrocytoma, choroid plexus carcinoma, glioblastoma, hemangiopericytoma, rhabdomyosarcoma, melanoma, mesenchymal hamartomas of the chest wall and the liver, and juvenile xanthogranuloma, with special consideration of blue rubber bleb nevus syndrome. MRI plays a significant role in further and better characterization of congenital tumors, leading to a correct diagnosis in many cases, which is crucial for pregnancy and neonatal management and psychological preparation of the parents. No diagnosis is impossible and can be absolutely excluded.

## 1. Introduction

Tumors are considered prenatal if detected in utero or in the first 3 months after birth [1,2]. Congenital malignant tumors constitute approximately 2% of pediatric malignancies [3]. The most frequent of them are presented in Table 1.

Tumors are usually detected by ultrasonography (US) and ruled out or confirmed and further characterized by magnetic resonance imaging (MRI), both in the pre- and postnatal period.

Diagnosis of a tumor in utero—as precisely as possible—has significant implications: from termination of pregnancy (depending on the country’s law system) to its continuation with prenatal treatment or ex utero intrapartum treatment (EXIT) (if possible); planning the time, mode, and place of delivery; planning postnatal management, e.g., early Cesarean section, implementation of chemotherapy, and—in more and more cases—surgery based on prenatal MRI without repeating the examination after birth [6,7]; and end-of-life anticipatory management. In our center, these important decisions are made by an interdisciplinary team of specialists with a radiologist among them. The International Society of Ultrasound in Obstetrics and Gynecology (ISUOG), in its guidelines concerning fetal MRI, encourages multispecialty teamwork by experts in the field of prenatal diagnosis in order to integrate the clinical picture, family history, and US and MRI findings to optimize patient care and provide the patient with the best counseling and management options [8]. It has been stated in the literature that interdisciplinary teams make better decisions than those made by single individuals [9].

Congenital tumors have been described in several publications [4,10,11,12,13] and also in quite numerous case reports. Most of the papers concern the most frequent teratomas. Lymphangiomas, hemangiomas, and cardiac rhabdomyomas are also frequently reported. The available English-language literature lacks comprehensive studies on the topic. There are mainly casuistic works on one type of cancer and case series. We also aim to make a strong case for this model of the diagnostic process, which implies reliance on the ALARA principle (As Low As Reasonably Achievable), compliance with the Image Gently campaign [14], and is based on methods that do not involve ionizing radiation (i.e., computed tomography, CT) in this youngest group of patients. The purpose of our study is also to present those more common but also rare, less frequently occurring tumors diagnosed based on fetal or/and postnatal MR examinations conducted in our Department of Diagnostic Imaging at the Institute of Mother and Child in Warsaw, in correlation with histopathologic findings if available and with particular emphasis on unique cases.

## 2. Materials and Methods

**Figure 1:**
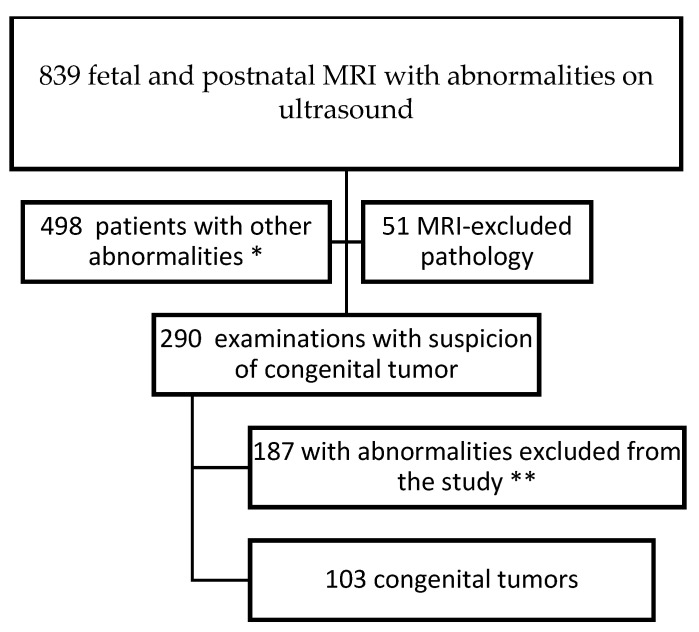
Selection of the study group. * brain malformations such as callosal agenesis, heterotopias, schizencephaly, holoprosencephaly, rhombencephalosynapsis, hydrocephalus, etc.; skeletal deformations; cardiac malformations; renal agenesis; hernias. ** congenital cysts, i.e., duplication cysts, ovarian cysts, adrenal cysts; CCAM; cystic lymphatic malformations; etc.

A total of 839 fetal and postnatal MRI studies performed in the first 3 months of life from the period 2006–2023 were retrospectively reviewed. In 290 cases, there was suspicion or diagnosis of a congenital tumor. After selection, 103 cases were more closely analyzed. The exclusion criteria were as follows: lymphatic malformations, simple cysts, and developmental disorders (such as duplication cysts, etc.) The flowchart is presented in Figure 1.

**Figure 2:**
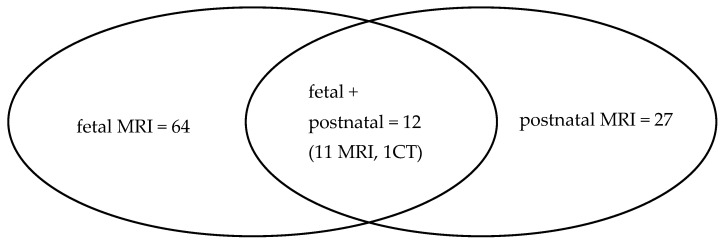
Fetal and postnatal diagnoses in the presented material.

MRI was performed—always after ultrasound as the first-line imaging study—with the use of two 1.5 T GE scanners: Signa HDxt and Signa Artist. The fetal MRI protocol consisted of the following sequences: SSFSE/T2WI, FIESTA, LAVA-FLEX, FSE/T1WI, DWI, and GRE EPI in three planes. Contrast medium was not administered to pregnant patients. The protocol of postnatal MRI was adjusted to the body area suspected for the pathology, with DWI and post-contrast T1-weighted images. The distribution of scans in the pre- and postnatal periods is illustrated in Figure 2.

Comparative analysis of signal intensity in different sequences allowed for the assessment of solid versus cystic tumors (SSFSE/T2WI, FIESTA), the presence of fat (LAVA-FLEX), calcifications or/and posthemorrhagic hemosiderin deposits (GRE EPI) or active bleeding, and diffusion restriction as an indicator of potential malignancy (DWI). The presence of fat was confirmed by suppression of its T1-high signal intensity (SI) on T1WI with fat saturation in the LAVA-FLEX sequence. Diffusion restriction is encountered in the DWI sequence when the random (Brownian) motion of water molecules in tissue is stopped by an obstacle (tumor) and is reflected by low values of the apparent diffusion coefficient (ADC). Hemosiderin deposits and/or calcifications are imaged as areas of low SI/lack of signal in the GRE EPI sequence in fetal MRI and older MRI studies and in the SWAN sequence in recent studies. In most cases, the widely accepted formula for the volume of an ellipsoid is used to calculate tumor volume: V = π/6 ⋅ (length) ⋅ (width) ⋅ (height).

This analysis received approval from the Bioethics Committee of the Institute of Mother and Child in Warsaw (opinion nr 20/2022).

## 3. Results

The diagnosis of a congenital tumor was established in 103 cases: 76 tumors were diagnosed based on fetal MRI between 20 and 37 gestational weeks (GW), and 27 were found after birth, from 1 day of age to 3 months of life.

Teratomas were the most common tumors in our dataset, mainly in the sacrococcygeal region (SCT), followed by cardiac rhabdomyomas and subependymal giant cell astrocytomas (SEGA) associated with TSC, and neuroblastomas. The group of less common tumors consisted of infantile fibrosarcomas, malignant rhabdoid tumors, mesoblastic nephromas and Wilms tumor, craniopharyngiomas, brain stem gliomas, desmoplastic infantile astrocytoma, choroid plexus carcinoma, glioblastoma, hemangiopericytoma, rhabdomyosarcoma, melanoma, mesenchymal hamartomas of the chest wall and of the liver, and juvenile xanthogranuloma, with special consideration of blue rubber bleb nevus syndrome.

Not all of the tumors had histopathological verification (five tumors were classified as teratomas, tumors related to Tuberous Sclerosis Complex (TSC), and brain stem gliomas) or feedback confirmation; however, when imaging was highly suggestive, these cases were included in the study. As far as TSC is concerned, a group of experts working on TSC with the EPISTOP consortium finally approved prenatal imaging as sufficient to diagnose the disease in 2021 [15]. Primary tumors of the brain stem are most often diagnosed on the basis of clinical and MRI findings, and a presumptive diagnosis of diffuse intrinsic pontine glioma (DIPG) based on classic imaging features is routinely employed in the absence of histologic verification, with biopsy recommended when the diagnosis is uncertain based on imaging findings [16]. Histological verification was achieved in 68 cases (66%) in our dataset.

The detailed results are presented in Table 2.

## 4. Discussion

Ultrasound is and will remain the undisputed first and primary imaging modality, both for fetuses, newborns, and the smallest children. Magnetic resonance imaging, with its multiplicity and variety of sequences, allows—by analyzing the same cross-sections in different sequences—very accurate analysis of the point of origin and nature of the tumor found on ultrasound. As a result, we were able to select only 103 cases for final analysis from a large original dataset (839 patients). On the one hand, computed tomography, the significant advantage of which is the speed of execution and accessibility, burdens the small patient with a dose of ionizing radiation, while on the other hand, it has much lower tissue resolution than MRI and, consequently, is a much less accurate diagnostic method. This causes us not to use this method in the diagnostic process. This is in line with the literature, which makes virtually no mention of fetal CT (except for bone dysplasias in some countries; however, this is not the subject of this paper) [17]. In the first months of postnatal life, the use of these methods most likely depends on the conditions of a given center; in ours, oncologists always request an MRI for the above-mentioned reasons.

**Figure 3:**
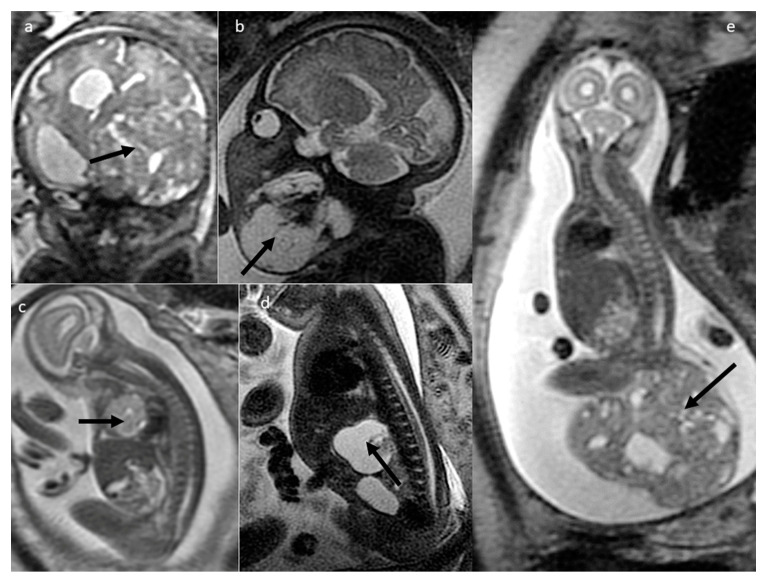
Different locations of teratomas (arrows) on fetal MRI (SSFSE/T2W and FIESTA images). (**a**) Intracranial; (**b**) facial; (**c**) anterior mediastinal; (**d**) abdominal; (**e**) sacrococcygeal (SCT type II).

Teratomas (Figure 3) constitute an important group of fetal tumors due to their frequency and potential consequences. In our study—as in the literature—sacrococcygeal teratoma (SCT) was the most common tumor (*n* = 25) and was diagnosed from 20 GW until 3 months of life. Most SCTs were solid and inhomogeneous (*n* = 16), and the remaining ones had a mixed (solid-cystic) nature (*n* = 3) or were purely cystic (*n* = 6). Calcified components or hemosiderin deposits within the tumors were found in eight cases (out of 13 for which T2*WI was acquired), and evident fat components were found in three cases. Tumors showed restricted diffusion in 12 out of 17 cases for which the DWI sequence was performed, always in a solid part.

According to the American Association of Pediatrics Surgical Section, SCT can be classified into four types [18]:

- External mass only
- Equal internal/external components (dumbbell shape)
- Primary location in abdomen or pelvis
- Entirely internal, no external components visible

In our study there were 6 cases of type I, 14 of type II, only one of type III and 2 cases of type IV. Treatment requires complete resection including coccyx [19].

Two children with large tumor volume exceeding 300 mL died soon after birth, ten patients are still under control after successful surgery, the remaining children were treated outside our Institute and their outcomes are unknown. 

Brain and head and neck teratomas were the second most numerous group of teratomas in the analyzed dataset, which complied with other authors’ statistics on the occurrence of these tumors [1,4]. In five fetuses, the tumor was located in the brain, causing significant mass effect. Calcifications were depicted in six cases, evident intratumoral fat was found in one case, and five tumors showed restricted diffusion. Death soon after birth in two cases was due to intracranial location in one case and compression of the airway in the other case. Tumors in the head and neck and chest regions were analyzed by the interdisciplinary team with the invited anesthesiologists due risk of airway compression after birth and in order to plan the EXIT procedure.

The remaining teratomas in our group (*n* = 8) were localized in the chest or abdomen: intra- and extraperitoneally, in the mediastinum, in the chest/abdominal wall, and in the umbilical cord. Those tumors also presented calcifications (*n* = 4), fat (*n* = 1), and restricted diffusion (*n* = 3).

**Figure 4:**
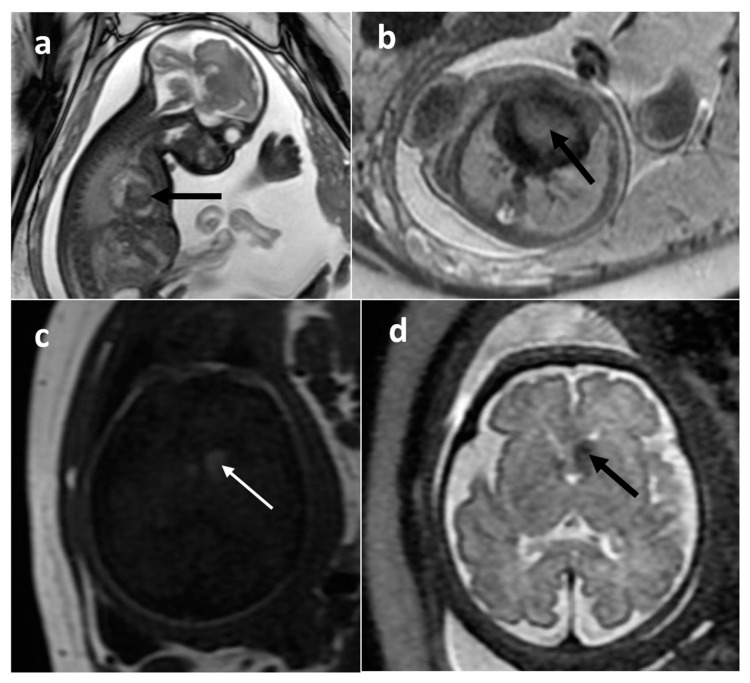
Tuberous sclerosis complex (TSC) on fetal MRI. Cardiac rhabdomyoma: (**a**) sagittal FIESTA; (**b**) axial SSFSE/T2. Subependymal giant cell astrocytoma (SEGA): (**c**) T1WI; (**d**) SSFSE/T2WI.

TSC-associated lesions were the second most numerous group of tumors in our study. Cardiac rhabdomyomas (Figure 4a,b) are the most common cardiac tumors diagnosed in the prenatal period and are strongly correlated with TSC [20]. In our study, all of the tumors were first diagnosed based on fetal echocardiography, and MRI was performed in search of the associated brain lesions [21]. Cardiac rhabdomyomas are typically hyperintense on T2WI. In the brain, among the many T2-hypointense subependymal nodules found in children with TSC, they matched the criteria of SEGA in six cases (Figure 4c,d), i.e., were located in the foramen of Monro and had more than 10 mm in diameter [22,23].

The critically important thing about TSC is the ability to diagnose it in fetal life based on imaging criteria (congenital tumors: cardiac rhabdomyomas and SEGAs constitute two major diagnostic criteria), making it possible to introduce early antiepileptic treatment in these children at a recommended time of less than 16 weeks of postnatal life [15].

Neuroblastoma (Supplementary Figure S1) is a malignant tumor of sympathetic chain primitive cells. In 20% of cases the diagnosis is established in the first 3 months of life or prenatally [24]. In the literature, it is described as solid or cystic [1]. Neuroblastoma is more commonly cystic in fetuses and solid in neonates. Adrenal glands are the most common location, followed by thoracic and neck sympathetic chain ganglia [24]. In our study, most tumors (*n* = 4/5) were localized in the adrenals, and one originated from the infrarenal retroperitoneal space. 

One baby was diagnosed antenatally at 33 GW, and three others had MRIs between day 5 and the 3rd month of life. Tumors were solid and regular; three of five presented mild to evident diffusion restriction, and two of five contained calcifications on T2*WI. They were hypo- to isointense or had mixed signal intensity on T1WI and hyperintense or with mixed SI on T2WI. Heterogenous hypointensity on T1WI and hyperintensity on T2WI is also described in the literature [25].

**Figure 5:**
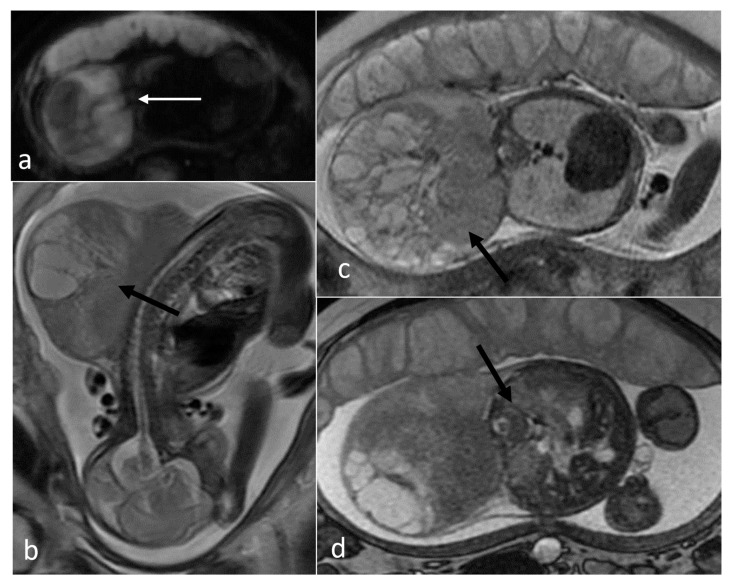
Infantile fibrosarcoma (arrows) in a fetus at a GA of 29 weeks. Huge heterogenous solid and cystic back mass infiltrating vertebral arches. (**a**) Axial DWI; (**b**) sagittal SSFSE/T2WI; (**c**) axial SSFSE/T2WI; (**d**) axial FIESTA.

Infantile fibrosarcoma (IFS) is the most common soft tissue malignant congenital tumor. It is less aggressive than other sarcomas (locally destructive) with low metastatic potential. Usually it arises in the extremities, rarely it can be also found in the back (as it was in our two cases), neck, or retroperitoneum [1,26]. In the first case, fetal MRI at 29 GW showed a large (423 mL), well-defined, heterogenous (solid/cystic) back mass with calcifications/blood products, mostly T2-hyperintense and iso-/hypointense on T1WI with diffusion restriction. The tumor destroyed vertebral arches and paraspinal muscles (Figure 5). There was no evidence of fat within it. The baby was born prematurely and died shortly after. IFS usually deforms bones and destruction is only seen sporadically, as in this case. In the second case of a 2-month-old child, a large (68 mm), paraspinal, intramuscular mass was found, exceeding from Th11 to L4, penetrating into the intervertebral foramina and extraperitoneal space, displacing and compressing the left kidney. On MRI, it showed numerous tortuous vessels, diffusion restriction, and heterogenous contrast enhancement (Supplementary Figure S2). Moreover, the tumor infiltrated the bone marrow of two ribs without cortical destruction or caused bone marrow edema.

**Figure 6:**
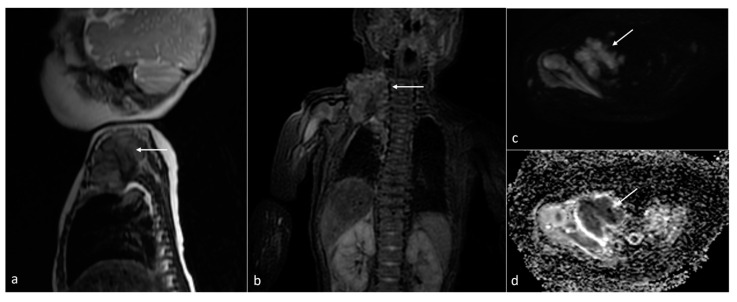
Malignant rhabdoid tumor (MRT) of the brachial plexus (arrows) with infiltration of the right clavicle, right lung, and muscles in a newborn girl. Inhomogeneous mass with low ADC values. (**a**) Sagittal T2WI; (**b**) coronal STIR; (**c**) axial DWI; (**d**) corresponding ADC map.

Malignant rhabdoid tumors (MRTs) are rare, aggressive tumors most often located in the renal region or intracranially (if affecting the CNS, they are commonly called atypical RTs). Tumors situated elsewhere in the body are even less common. Independent of the location, the outcome is dismal compared to that of other congenital tumors [27]: fast progression, metastatic potential, and no response to treatment—chemotherapy or surgery. In our dataset, there was a neonate with a fast-growing, hard tumor on the right brachial plexus, first noted by the parents at the age of 10 days. The first MRI performed outside our institution showed a heterogenous mass occupying the brachial plexus, clavicle, and upper pole of the lung on the right, with enlargement of local lymph nodes (Figure 6). After the diagnosis had been established, the girl started chemotherapy but died at the age of 6 months due to tumor progression, superior vena cava syndrome, and pulmonary insufficiency. In a second case, a 3-month-old girl with a tumor on an arm and the first diagnosis of Ewing sarcoma (very rare in the fetal or neonatal period [28,29]), the final pathologist’s report after additional genetic tests was MRT. Our MRI performed after biopsy and during the first weeks of chemotherapy showed a mostly soft tissue mass invading the humerus that was heterogenous with diffusion restriction and strong contrast enhancement. After chemotherapy, the child underwent partial amputation of the humerus with arthroplasty. The outcome to date is very good—the girl is now 6 years old with no relapse of the disease. 

Mesoblastic nephroma (Boland’s tumor) is the most common renal tumor in the prenatal period and early infancy up to 6 months of life. Polyhydramnios due to increased urine production may be found during pregnancy in up to approximately 70% of cases. Although the classic type can be treated surgically with good results, the cellular variant has a higher risk of recurrence and even can metastasize [30]. Boland’s tumor is predominantly solid, extending from or being found within the renal parenchyma, and usually does not invade renal vessels or the inferior vena cava. However, the cellular variant more often presents with cystic components (hemorrhagic or necrotic). Restricted diffusion has been reported in the solid parts of the mass [31]. One of our patients had fetal MRI at 31 GW (Supplementary Figure S3) and the second one underwent MRI on the second day of life. Both tumors followed the above mentioned characteristics, and the claw sign confirming renal origin was seen in both cases. Mild contrast enhancement within the tumor was seen in the newborn. 

**Figure 7:**
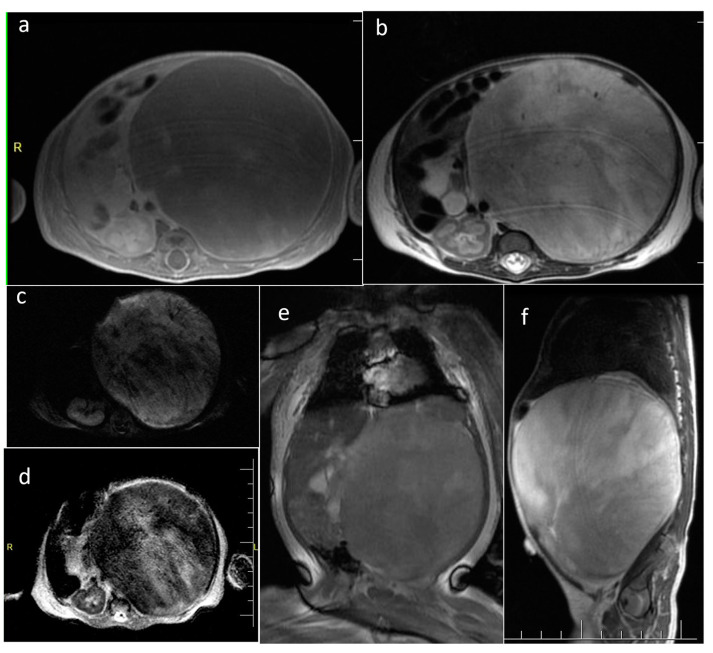
Wilms tumor arising from the left kidney in a newborn—encapsulated, heterogenous with necrosis, mass effect, contrast enhancement and restricted diffusion in solid parts. (**a**) Axial CE-T1WI; (**b**) axial T2WI; (**c**) DWI; (**d**) corresponding ADC map; (**e**) coronal FIESTA; (**f**) sagittal T2WI.

Wilms tumor should be considered in differential diagnosis of congenital renal tumors, but it is far less frequent than mesoblastic nephroma [30]. We included one patient with Wilms tumor who underwent MRI on the first day of life (Figure 7). The tumor arising from the left kidney was about 9 cm long, well-defined, encapsulated, heterogenous with a cystic component (necrosis), evident mass effect, and showed contrast enhancement and restricted diffusion in the solid parts. No infiltration of the adjacent structures was found. The baby underwent surgery and chemotherapy. No metastatic disease was stated and the child is still disease-free and the condition is under control after successful treatment. On MRI, it is hard to differentiate mesoblastic nephroma and Wilms tumor. The latter has its peak incidence between 2 and 4 years of age and is not commonly seen in the neonatal period. In this case, the histologic report facilitated the final diagnosis.

Craniopharyngiomas are histologically benign but may cause severe damage to the optic nerves and brain parenchyma. They represent as many as 7% of all congenital tumors of the central nervous system [13]. They typically arise from the Rathke pouch, which raises suspicion of this particular diagnosis, although teratoma should also be taken into account in such cases. Our study included two cases of craniopharyngioma imaged at 28 and 33 GW (Supplementary Figure S4) [32]. US depicted the hyperechogenic masses, but their relationship to the optic chiasm and brain stem could only have been characterized on MRI. On MRI, the lesions were inhomogeneous and showed a mass effect. 

**Figure 8:**
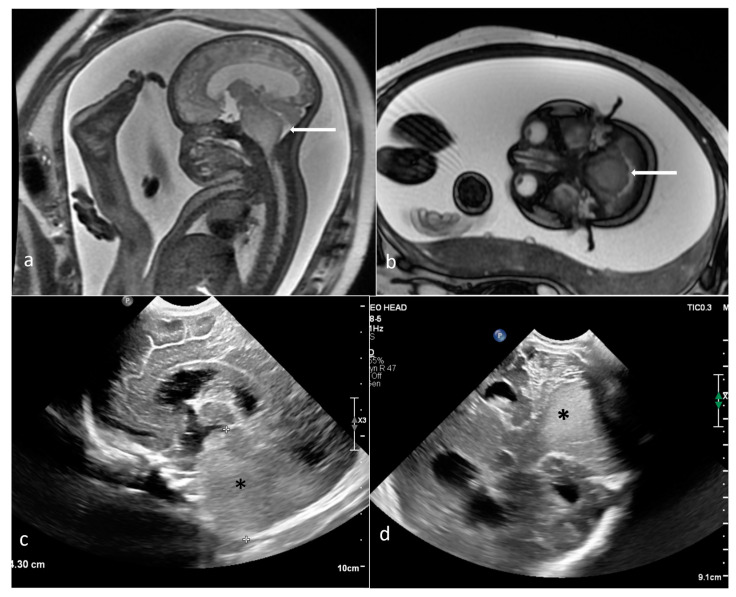
Diffuse brain stem glioma. Fetal MRI (arrows): (**a**) sagittal T2WI; (**b**) axial FIESTA. (**c**,**d**) US images of a brain stem mass reaching through the mastoid fontanelle after birth (asteriks).

A total of 10–15% of brain tumors in children arise from the brain stem, with the majority being gliomas, but they are rarely encountered as congenital. Tumors of the brain stem carry exceptionally poor prognosis associated with the involvement of crucial areas managing vital functions in this location and limited forms of therapy. When a mass suspected of being glioma is found in the brain stem, with all MR images characteristic and specific for glioma, it is advised to withdraw from biopsy [33,34]. We came across two examinations that revealed brain stem pathology. In the first case, it was a healthy newborn boy with a mass found incidentally on routine transfontanel ultrasound 2 days after birth. MRI showed an inhomogeneous, eccentric mass with small cysts and calcifications/hemorrhagic foci enlarging the brain stem, compressing the 4th ventricle and cerebellum with neither contrast enhancement nor restricted diffusion (Supplementary Figure S5). The appearance was consistent with brain stem glioma. The child was referred to the neurosurgical clinic, but neither biopsy nor other treatment was implemented. The boy was entrusted to the care of hospice. Follow-up MRI showed progression and radiologically confirmed the unfavorable prognosis. In the second case, there was prenatal suspicion of cerebellar tumor on US. MRI at 35 GW showed a heterogenous pathological mass expanding the pons and medulla oblongata. The diagnosis was sustained on the basis of transfontanel ultrasound after birth (Figure 8). The management was similar to the previously described one. The child passed away in the second month of life.

Desmoplastic infantile astrocytoma (DIA) in a fetus scanned at 34 GW resembled a meningioma on MR images as totally solid, well-circumscribed, T1-hyperintense, T2-hypointense, located in the middle cerebral fossa in contact with the dura matter, and seemed to be an extra-axial mass (Supplementary Figure S6). Good delineation from the surrounding brain tissue and no visible edema suggested a benign tumor. Also, on the first day of postnatal life, the tumor behaved like a meningioma on MRI with homogenous strong contrast enhancement and no cystic elements [5]. DIA is an extremely rare solid astrocytic tumor accompanied by a cyst/cysts and involving the superficial cerebral cortex and leptomeninges [35]. It is usually benign and has a favorable prognosis, but also usually has a malignant MR appearance [36]. Our case was completely different: entirely solid, without cysts, with a benign, meningioma-like appearance, and—to our knowledge—the first described in the prenatal period on MR imaging. To our knowledge, it is also the first described case of completely solid DIA in the literature. It seems likely that our case represents the natural history of this tumor, which is solid at the beginning and produces cysts with time.

Choroid plexus papilloma is a benign and rare tumor that accounts for 0.4–0.6% of fetal intracranial tumors. In 20% of cases, it turns out to be choroid plexus carcinoma (CPCa) [37], so CPCa is a very rare fetal malignancy and represented once in our dataset in a fetus at 34 GW [5]. With its large dimensions, inhomogeneity (solid, cystic, and hemorrhagic elements), and mass effect, it was diagnosed as a malignant tumor (Supplementary Figure S7). It was indistinguishable on the basis of MR appearance from another malignant tumor in the study group—glioblastoma multiforme (GBM), which was imaged at 26 GW and showed strikingly low ADC values (Supplementary Figure S8) [38]. 

On fetal MRI the diagnosis of malignant brain tumors was only established in both cases with immature teratoma in differentials but without the specific suggestion of a tumor type as opposed to the cases of craniopharyngioma. US showed irregular, hypervascular masses in the brain and MRI better depicted their extent and relationship to the intracranial structure. Both babies were born prematurely by Caesarean section; the one with CPCa died during surgery due to massive hemorrhage, while the one with GBM died 90 min after CC due to significant hemodynamic insufficiency.

Glioblastoma is one of the rarest congenital brain tumors and uncommon in the prenatal period [38,39]. The group of high-grade astrocytomas consists of heterogenous, fast-growing lesions with poor prognosis and low global survival rate [13]. According to the newest 2021 WHO classification of brain tumors [40], the entity “glioblastoma multiforme” no longer exists. Our patient was born in 2013, so the tumor was assessed on the basis of earlier classification. 

**Figure 9:**
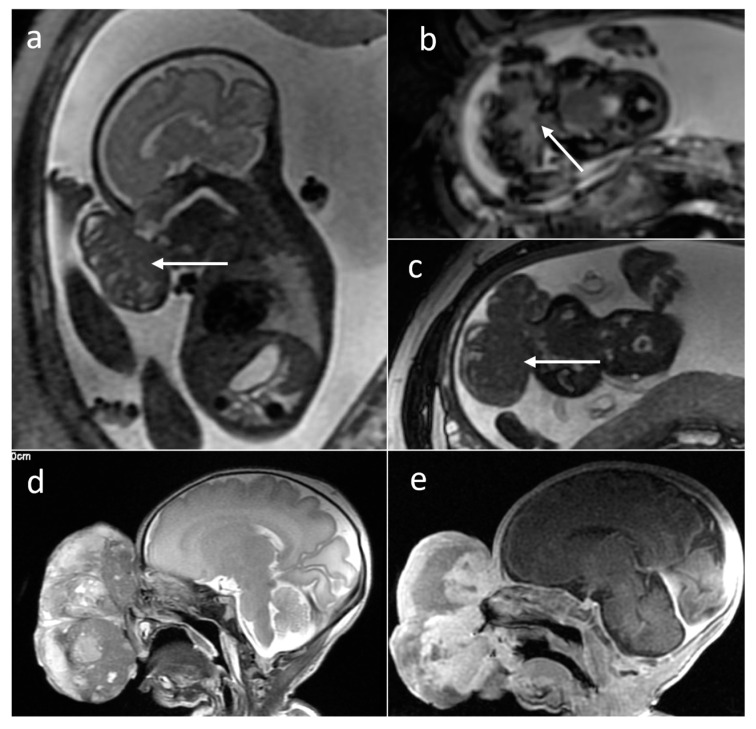
Extra-pleural hemangiopericytoma/solitary fibrous tumor in a fetus (arrows). A mass of mixed signal intensity deriving from the face, with peripheral deposits of hemosiderin. (**a**) Sagittal SSFSE/T2WI; (**b**) axial GRE EPI; (**c**) axial FIESTA. Postnatal MRI: (**d**) sagittal T2WI; (**e**) CE-T1WI+Gd showing inhomogeneous contrast enhancement.

Extra-pleural hemangiopericytoma/solitary fibrous tumor (HPC/SFT) is commonly associated with intracranial location and similarity to meningioma, from which it should be differentiated. Our single case of HPC/SFT was found in a much rarer head and neck location as a huge, inhomogeneous, extrafacial mass with irregular borders, which was considered on US as pedunculated, deriving from the nose, and most likely representing hemangioma. Fetal MRI performed at 29 GW (Figure 9) did not show any peduncle, the nasal cavity was tumor-free, and the mass was deriving from the face, which was confirmed on visual inspection and MRI on the first day of postnatal life. The neonate was operated on 2 days later, the tumor was successfully resected, and the final pathological diagnosis was neonatal-type HPC/SFT. 

Fetal HPC/SFT is reported a few times in PubMed; these publications are case reports and concern intracranial localization in fetuses [37,41,42]. We have not found reports on tumors growing exophytically from the face, although the cheek was mentioned once as a point of possible origin of the tumor [43]. The child is now 7 years old, and the condition is still under control with no signs of recurrence.

Rhabdomyosarcoma (RMS) is an aggressive, malignant soft tissue tumor. It accounts for 4–8% of malignancies in children under 15 years of age [44], but it is rarely encountered as congenital. RMS can be found in different parts of the body, but the most common localization is the head and neck and genitourinary system [4]. 

**Figure 10:**
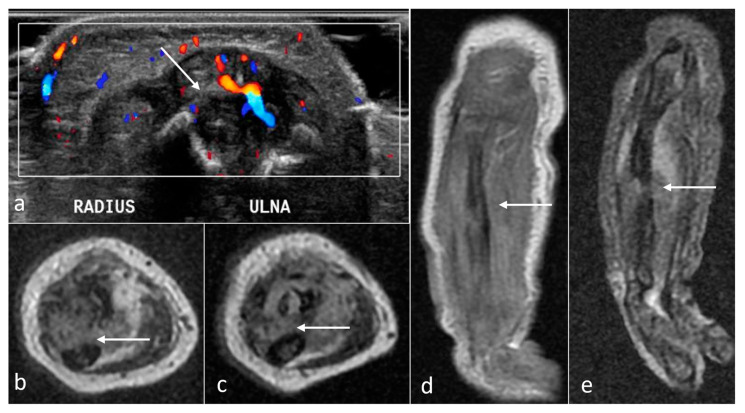
Rhabdomyosarcoma (arrows) in a newborn with a soft tissue mass in a forearm, adjacent to the ulna and radius, causing periosteal reaction, with restricted diffusion and inhomogeneous, moderate contrast enhancement. (**a**) US transverse section; (**b**,**c**) axial T2WI; (**d**) coronal CE-T1WI; (**e**) coronal STIR.

In our dataset, there was one patient with this malignancy—a boy with a fast-growing forearm mass, diagnosed right after birth, which turned out to be embryonal spindle cell-type RMS without metastases. MRI performed in the first week of life (Figure 10) revealed a soft tissue mass adjacent to the ulna and radius, causing a periosteal reaction. In agreement with the commonest imaging features, it presented SI isointense to the muscle on T1WI and hyperintense on T2WI. The tumor was 4 cm in the largest dimension and showed restricted diffusion and inhomogeneous, moderate contrast enhancement. The child underwent chemotherapy and surgery and is now 6 years old, with the condition still under control with no recurrence.

**Figure 11:**
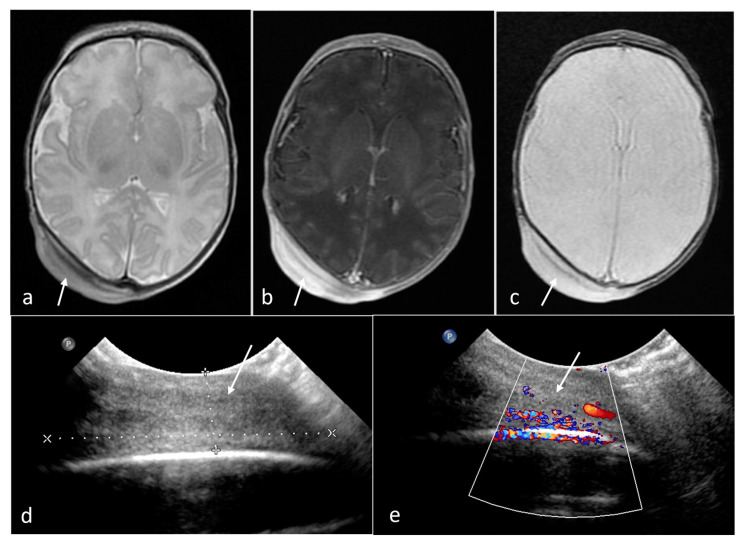
Malignant melanoma in a neonate (arrows). Solid, firm, occipital soft tissue lesion with strong contrast enhancement, without infiltration of the skull bones. (**a**) axial T2WI; (**b**) axial CE-T1WI; (**c**) axial GRE/T2*WI; (**d**,**e**) US sections.

In one case, we encountered particular diagnostic difficulties. A pathologic mass on the head was noticed right after birth. On US, it was hyperechoic and rather well-demarcated with few vessels on color doppler images. On MRI, it was a homogenous, solid mass in the soft tissues of the scalp in the parietal region, 40 mm in diameter and 11 mm thick. It showed strong contrast enhancement (Figure 11). After biopsy, the initial diagnosis was reticulohistiocytoma, but the tumor did not respond to treatment; additional biopsy was conducted and the final diagnosis was amelanotic malignant melanoma. The child passed away in the 2nd year of life. 

Congenital melanomas are very rare. According to Tariq et al. [45], only ten cases of congenital scalp malignant melanoma are described in the literature, of which six arose from congenital melanocytic nevi. As in our patient, this pathology carries a poor prognosis.

Hepatoblastoma is the most common congenital primary hepatic malignancy. It can carry a poor prognosis due to hemorrhage risk, but the actual outcome depends on the stage of the disease, which is established on the basis of resectability features and the presence of metastases [4,46].

A boy with a prenatally diagnosed hepatic mass on US underwent postnatal MRI, which revealed a typical tumor appearance, i.e., solid, inhomogeneous, well-defined mass (pseudocapsule) with the spoke-wheel sign, numerous hemorrhagic foci/calcifications, and moderate contrast enhancement (Supplementary Figure S9). Additional tests showed no signs of metastatic disease. Treatment consisted of chemotherapy and surgery. The child is now 6 years old, and the condition is still under control with no recurrence.

Among tumors arising from fibrous tissue, benign infantile myofibroma or myofibromatosis when multiple myofibromas are present, according to literature, may have some nonspecific features on MRI, such as low or intermediate signal intensity on T2W images and extension along fascial planes that support the diagnosis of myofibroblastic tumor [47].

In a 2-week-old girl, a solid lesion extending from the pelvis through the left buttock to the thigh was found on MRI (Supplementary Figure S10). The mass was avidly enhancing and presented all of the features mentioned above. The child is still under observation, without surgical treatment. 

**Figure 12:**
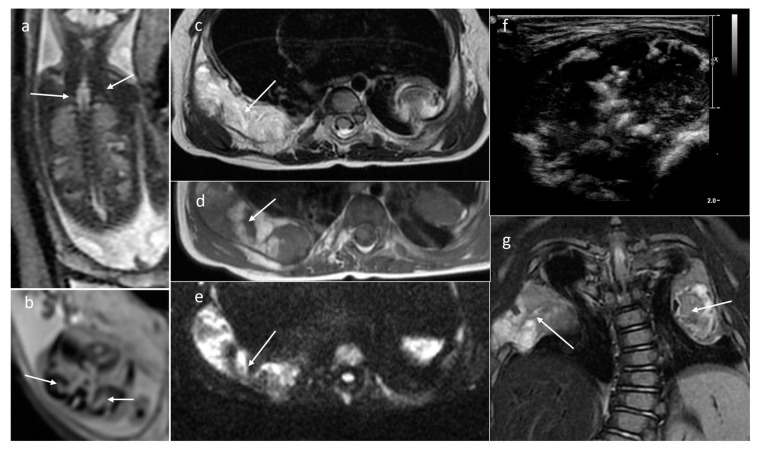
Mesenchymal hamartomas of the chest wall. Bilateral lesions of the ribs in a fetus. (**a**) Coronal SSFSE/T2WI; (**b**) axial GRE EPI. Follow-up MRI after birth. (**c**) Axial T2WI; (**d**) axial T1WI; (**e**) DWI; (**f**) US transverse section; (**g**) coronal T2WI.

Mesenchymal hamartoma of the chest wall (MHCW) is a rare tumor that typically presents in infancy as a chest wall mass with or without respiratory distress and with marked rib deformity. It arises from the ribs and consists of partially mineralized focal overgrowths of normal skeletal elements with no malignant potential. There may be some hemorrhagic cavities within the lesions, leading to aneurysmal bone cyst formation. These cystic elements were mainly seen on T2WI, but costal deformations were clearly depicted in a fetus of 23 GW in the GRE EPI sequence (Figure 12) and the tentative diagnosis of MHCW was established, while these masses were mistaken for lung lesions on prenatal ultrasound. The appropriate diagnosis is of the utmost significance, as the prognosis and treatment differ in both kinds of pathology, with a definitely better prognosis in the case of chest wall lesions. After birth, an X-ray confirmed the diagnosis and since the masses did not cause any respiratory distress, the newborn was discharged from our Institute with a recommendation for further inspection. At the age of 1 year, MRI was performed and revealed progression of the lesions within the ribs with atelectasis of the upper lobe of the right lung ad of the posterior segment of the lower lobe. There was scoliosis and deformation of the chest. Multiple smaller lesions were found in the vertebral column and one was found in the sternum. This radiological appearance further confirmed the primary antenatal diagnosis, as this specific distribution of these rare tumors has been described in the literature [48].

We also dealt with congenital hepatic mesenchymal hamartoma (Supplementary Figure S11). This tumor is reported together with hemangioma as the most frequent fetal and neonatal tumor of the liver, with heterogenous cystic and solid components and myxoid or fibrous stroma [46]. In our case, MRI was performed on a fetus of 30 GW and revealed a large, inhomogeneous, cyst-like mass deriving from lower part of the liver, which was later confirmed to be mesenchymal hamartoma. 

**Figure 13:**
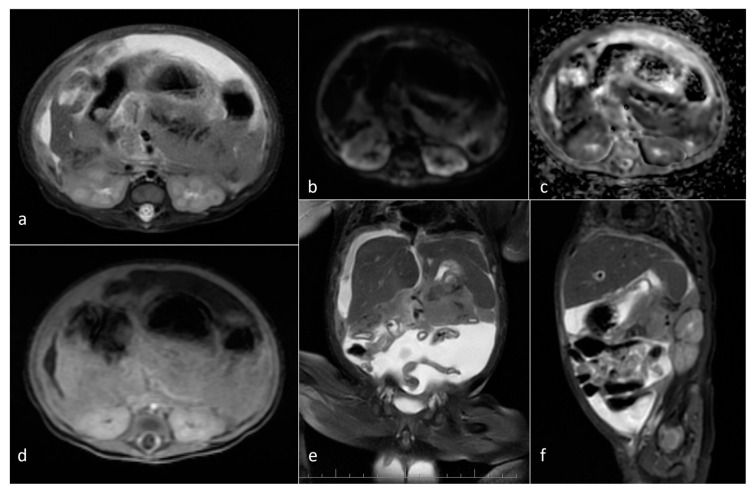
Juvenile xanthogranuloma. MRI of a neonate with irregular, solid, enhancing masses that are difficult to separate from internal organs. Increased volume of fluid in the abdominal cavity. (**a**,**e**,**f**) T2WI+fatsat: axial, coronal, and sagittal, respectively; (**b**) DWI; (**c**) corresponding ADC map; (**d**) axial CE-T1WI+fatsat.

A 3-week-old boy with hepatosplenomegaly and intra-abdominal masses accompanied by ascites was admitted to our center with suspicion of systemic infiltrative juvenile xanthogranuloma (JXG) after biopsy/sampling of fluid from the abdominal cavity. JXG is a rare disease, classified as non-Langerhans cell histiocytosis, which mostly invades the skin and subcutaneous tissue, whereas systemic involvement is rather unique. The mother suffered from SARS-CoV-2 infection 3 weeks before delivery and no sooner after this infection, fetal hepato/splenomegaly and ascites were stated on fetal US. MRI of the neonate (Figure 13) depicted irregular, solid, enhancing masses with restricted diffusion that were difficult to separate from the internal organs (adrenal glands, stomach, spleen, and pancreas) and also located along large vessels (enlargement of lymph nodes). There are only a few reports of systemic disease but none of them presented the involvement pattern similar to that in our patient. He et al. described a patient with multiple subcutaneous nodular lesions on the trunk and extremities, multiple bilateral peripheral pulmonary nodules, and also abdominal changes, but the hypodense lesions in the liver and left kidney were completely different from our patient [49,50].

**Figure 14:**
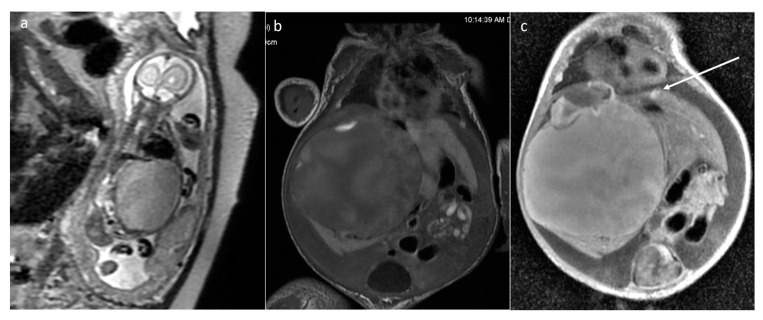
Another case of BRBNS in a fetus. (**a**) Coronal SSFSE/T2WI. Postnatal MRI of the intra-abdominal mass with peripheral contrast enhancement and hemorrhagic foci (bright spots on T1-weighted images); (**b**) coronal T1WI; (**c**) coronal CE-T1WI+fatsat; (arrow points at another small lesion in the left liver lobe).

Finally in our material there are two cases of an unusual condition that was assessed on MRI as tumors—blue rubber bleb nevus syndrome (BRBNS, Bean syndrome). The syndrome is mentioned in a few non-fetal papers concerning facial region [51], but no publication was found regarding fetal MRI. BRBNS is a rare condition characterized by the presence of venous malformations in the skin, gastrointestinal tract (small intestine and distal large bowel), and other parts of the body, and carries the potential for serious bleeding. It usually presents soon after birth but—as in our cases—may also be discovered prenatally [21]. In the first case, the tumor was described on US as a mass of mixed echogenicity with moderate vascularization, deriving from the oral cavity. MRI performed at 35 GW showed the tongue as the point of origin of the mass, which was correct, but the tumor was mistaken for teratoma (Supplementary Figure S12) due to the erroneous assumption that T1-hyperintensity reflects fat and T1-fat-saturated images were not obtained. Nevertheless, lack of macroscopic fat within a tumor does not rule out teratoma. The other case of BRBNS in our dataset presented as a huge cyst in the abdomen and pelvis of a fetus at 25 GW. On US, multiple liver cysts were described, while MRI (Figure 14) showed a single cystic lesion filling almost the entire abdomen and pelvis. Caesarean section was performed. After birth, numerous skin nodules were found on visual inspection and numerous contrast-enhancing nodules were shown on MRI in the internal organs (liver, diaphragm, chest wall) in addition to the single abdominal cystic lesion. The condition was initially mistaken for a malignant tumor with metastatic spread, which was suggested on postnatal MRI. The neonate was operated on and died during surgery due to massive hemorrhage.

Prenatal recognition of a tumor implies changes in pregnancy and neonatal management depending on the time of diagnosis, the country’s legislation, and the religious/ethical beliefs of the parents. In the setting of a tertiary referral center with an interdisciplinary team for fetal diagnosis and therapy, it influences many practical issues, such as the choice of place, time, and mode of delivery, pre- or postnatal treatment, and genetic counseling. In every case, the team considered these issues individually, coordinating the activities of various specialists. The admission of a newborn to the appropriate clinic dealing with tumors that were not cured at our Institute, like brain tumors, was also agreed upon in advance, giving a sense of security to the parents exposed to the enormous stress associated with the baby’s disease. Even if the final diagnosis cannot be established, distinction between a malignant and benign tumor is of great psychological importance to the parents: calming in the case of benign lesions and preparing for a poor prognosis in the case of malignant disease. MR images are easier and more understandable to parents, usually non-physicians, than US images. The larger field of view than on US and the ability to show the whole situation in one image make this method more useful in difficult sessions with parents. Surgeons and oncologists also prefer the global view of the abnormalities provided by MRI [17,52], rely mainly on MRI, and always refer our youngest patients to MRI after ultrasound before making decisions.

Our study’s strength is a large dataset not limited to a single organ, body part, or tumor type. Another strength is its rich iconography, which can assist in these rare and diagnostically difficult clinical situations. Nevertheless, it also has a significant limitation of being only qualitative, with only a few entities with higher numbers of cases. These, in turn, as mentioned in the Introduction, are best described in the existing literature. In the long run, with further technical and application development of MRI and after collecting more cases, we hope to further confirm the superiority of MRI to US in diagnosis, guiding treatment, and prognostication of congenital tumors.

## 5. Conclusions

The significant role of MRI in our dataset was—as stated in the Introduction—further and better characterization of congenital tumors. MRI correctly depicted SCTs, brain masses as benign (DIA) or malignant (CPCa and GBM), and correctly diagnosed specific tumor types: SEGA and craniopharyngioma. In four cases, MRI aptly identified the point of origin of the tumors: the brain stem and not the cerebellum, the tongue in BRBNS, the surface of the face and not the nasal cavity (SFT), and the ribs and not the lungs (MHCW). 

Our dataset reminds us of the need to remain humble in medicine, here in diagnostic imaging, in which there are no impossible and absolutely excluded diagnoses, e.g., due to the patient’s age or tumor location. It also makes us aware again of how difficult it is to determine the prognosis and counsel the parents of an unborn child.

## Figures and Tables

**Table 1:** The incidence of the most frequent congenital tumors [4,5].

Tumor Type	Incidence (%)	Additional Statistics
Extracranial teratoma	23–29	45% SCT
Neuroblastoma	22–30	90% adrenal gland
Soft tissue	8–12	Infantile fibrosarcoma most common
CNS	6–10	50% teratoma
Renal	5–7	66% mesoblastic nephroma
Hepatic	5	hemangioma 60% mesoblastic hamartoma 23% hepatoblastoma 16% (excluding metastases)
Thoracic	3	cardiac rhabdomyoma (TSC) 78% teratoma 18%

**Table 2:** Details of the imaging appearance of the tumors in the analyzed dataset.

Tumor	Localization	Number of Patients	Volume (cm^3^) Mean [Min–Max]	Diffusion Restriction (ADC Values Min–Max × 10^−6^ mm^2^/s)	Solid/Cystic	Hemosiderin Deposits or Calcifications on T2*	Contrast Enhancement
Teratoma	sacrococcygeal	25	268 [2.5–1145.165]	yes (13/17) [918–1661)	cystic (6) mixed (19)	yes 8/13	yes (3/4)
	head and neck	13	176.1 [1.78–877.11]	yes (9/13) (796–1294)	solid (3) mixed (8) cystic (2)	yes (8/13)	yes (1)
	thorax and abdomen	8	32 [1.49–134.98]	yes (3/6)	mixed (3) solid (3) cystic (2)	yes (4/5)	yes (1)
Rhabdomyoma	heart	22	5.08 [0.1–22]	no	Solid	No	N/A
Subependymal giant cell astrocytoma	foramen of Monro	6	0.74 [0.59–0.89]	no	Solid	yes (2/2)	N/A
Neuroblastoma	adrenal gland/infrarenal space	5	11.9 [2.6–24.2]	yes (3/5) (754–1200)	solid/mixed	yes (2/5)	yes (3/3)
Infantile fibrosarcoma	soft tissues of the back	2	235 [47–423]	yes (2/2) (1025–1030)	solid (1) mixed (1)	yes (1/1)	yes 1/1
Malignant rhabdoid tumor	soft tissues in upper right chest, soft tissues of left arm	2	33.8 [20.5–47]	yes (2/2) (638–791)	Solid	no (0/1)	yes 1/1
Mesoblastic nephroma	kidney	2	98.4 [42.9–153.9]	yes (1100–1310)	Solid	0/2	yes (1/2)
Wilms tumor	kidney	1	366.9	yes (548)	Mixed	N/A	yes (solid part)
Craniopharyngioma	sella turcica/suprasellar	2	34.15 [24.8–43.5]	yes (1/2) (548)	Mixed	no (0/1)	N/A
Brain stem glioma	brain stem	2	[12.5–18.41]	no (1266–1430)	microcystic/solid	1/2	0/1
Desmoplastic infantile astrocytoma (DIA)	right middle cranial fossa	1	52.7	N/A	Solid	N/A	N/A
Choroid plexus carcinoma	left cerebral hemisphere	1	182	N/A	Mixed	N/A	N/A
Glioblastoma	right cerebral hemisphere	1	140	yes (492)	Mixed	yes	N/A
Extra-pleural hemangiopericytoma/Solitary fibrous tumor (HPC/SFT)	nasal region	1	159.1	yes (1197)	Solid	yes	yes
Rhabdomyosarcoma	forearm	1	7	yes (885)	Solid	N/A	yes
Melanoma	scalp	1	17.5	yes (1230–1480)	Solid	No	yes
Hepatoblastoma	liver	1	282	yes (770)	mixed	No	yes (solid part)
Infantile myofibroma	left buttock and thigh, pelvis	1	461	yes (1056)	Solid	No	yes
Mesenchymal hamartoma	liver	1	157	N/A	Cystic	N/A	N/A
	chest wall	1	2.67	yes (1450)	Solid	yes	N/A
Juvenile xanthogranuloma (JXG)	abdomen	1	irregular shape	no	Cystic	N/A	yes
Blue rubber bleb nevus syndrome/Bean syndrome (BRBNS)	abdomen, tongue	2	141 [124–158]	no	Solid	1/2	N/A

## Data Availability

The data presented in this study are available upon request from the corresponding author. The data are not publicly available due to privacy.

## Supplementary Materials

The following supporting information can be downloaded at: https://www.mdpi.com/article/10.3390/cancers16010043/s1, Figure S1. Neuroblastoma in two different neonates. Mixed tumor of the left adrenal gland in one baby: (a) axial DWI; (b) coronal T2WI. Solid tumor of the right adrenal gland in another baby: (c) axial CE-T1WI with inhomogeneous contrast enhancement; (d) coronal STIR. Figure S2. Infantile fibrosarcoma. A 2-month-old girl with paraspinal, intramuscular mass with numerous tortuous vessels, diffusion restriction, and heterogenous contrast enhancement, penetrating into the intervertebral foramina and infiltrating the extraperitoneal space. (a) Axial STIR; (b) axial native T1WI; (c) ADC map; (d) axial CE-T1WI+fatsat, part of dynamic sequence. Figure S3. Mesoblastic nephroma in a 31-GW fetus. Homogeneous tumor following the signal intensity of a normal kidney on all sequences. (a) Axial SSFSE/T2WI; (b) axial DWI; (c) axial FIESTA; (d) coronal FIESTA; (e) coronal SSFSE/T2WI; (f) sagittal SSFSE/T2WI. Figure S4. Craniopharyngioma causing hydrocephalus on fetal MRI. (a) Axial DWI; (b) axial GRE EPI; (c) axial SSFSE/T2WI; (d) coronal FIESTA; (e) sagittal SSFSE/T2WI. Figure S5. Diffuse brain stem glioma in a newborn with small cysts and calcifications or hemorrhagic foci. (a) Sagittal 3D/FIESTA; (b) axial T2WI; (c) coronal CE-BRAVO/3D/T1WI; (d) axial SWI. Figure S6. Desmoplastic infantile astrocytoma (DIA) in a 34-GW fetus. A solid, well-circumscribed mass resembling a meningioma. (a, c, d) SSFSE/T2WI in axial, sagittal, and coronal planes, respectively; (b) axial T1WI. Figure S7. Choroid plexus carcinoma in a fetus at the GA of 34 weeks (a–c) Large, inhomogeneous tumor with solid, cystic, and hemorrhagic elements and mass effect, causing hydrocephalus. (a, b) Axial FIESTA; (c) coronal FIESTA; postnatal CT. Figure S8. Glioblastoma at 26 GW. Heterogenous mass with solid and cystic components with GRE/T2* hypointense areas (calcifications and/or blood products), significant mass effect, and low ADC values. (a) Coronal SSFSE/T2WI; (b) axial FIESTA; (c) sagittal SSFSE/T2WI; (d) DWI; (e) corresponding ADC map. Figure S9. Hepatoblastoma. A newborn boy with prenatally diagnosed hepatic mass—a solid, inhomogeneous, well-defined tumor with a spoke-wheel appearance and numerous GRE/T2* hypointense foci (blood products/calcifications). (a) Axial T2WI; (b) axial T1WI; (c) sagittal T2WI; (d) coronal T2WI. Figure S10. Infantile myofibromatosis. A 2-week-old neonate with a low signal mass on both T1- and T2-weighted images, extending from the pelvis through the left buttock to the thigh. (a) Coronal T2WI; (b) coronal T1WI; (c) axial CE-T1WI; (d) coronal CE-T1WI+fatsat FS; (a’) follow-up MRI in 16th month of life, coronal T2WI. Figure S11. Mesenchymal hamartoma of the liver in a fetus. Large, intra-abdominal, apparently cystic mass deriving from the lower part of the liver. (a, c, d) Oblique SSFSE/T2WI; (b) coronal T1WI. Figure S12. Blue rubber bleb nevus syndrome (BRBNS) in a fetus. A mass deriving from the tongue. (a) Axial GRE EPI; (b) sagittal FIESTA; (c) axial FIESTA; (d) sagittal SSFSE/T2WI.
