# Supplementary figures and images for: Congenital Tumors—Magnetic Resonance Imaging Findings with Focus on Rare Tumors
Source: Cancers (Basel). 2023 Dec 20;16(1):43. doi: 10.3390/cancers16010043 (PMC10778132; doi:10.3390/cancers16010043)

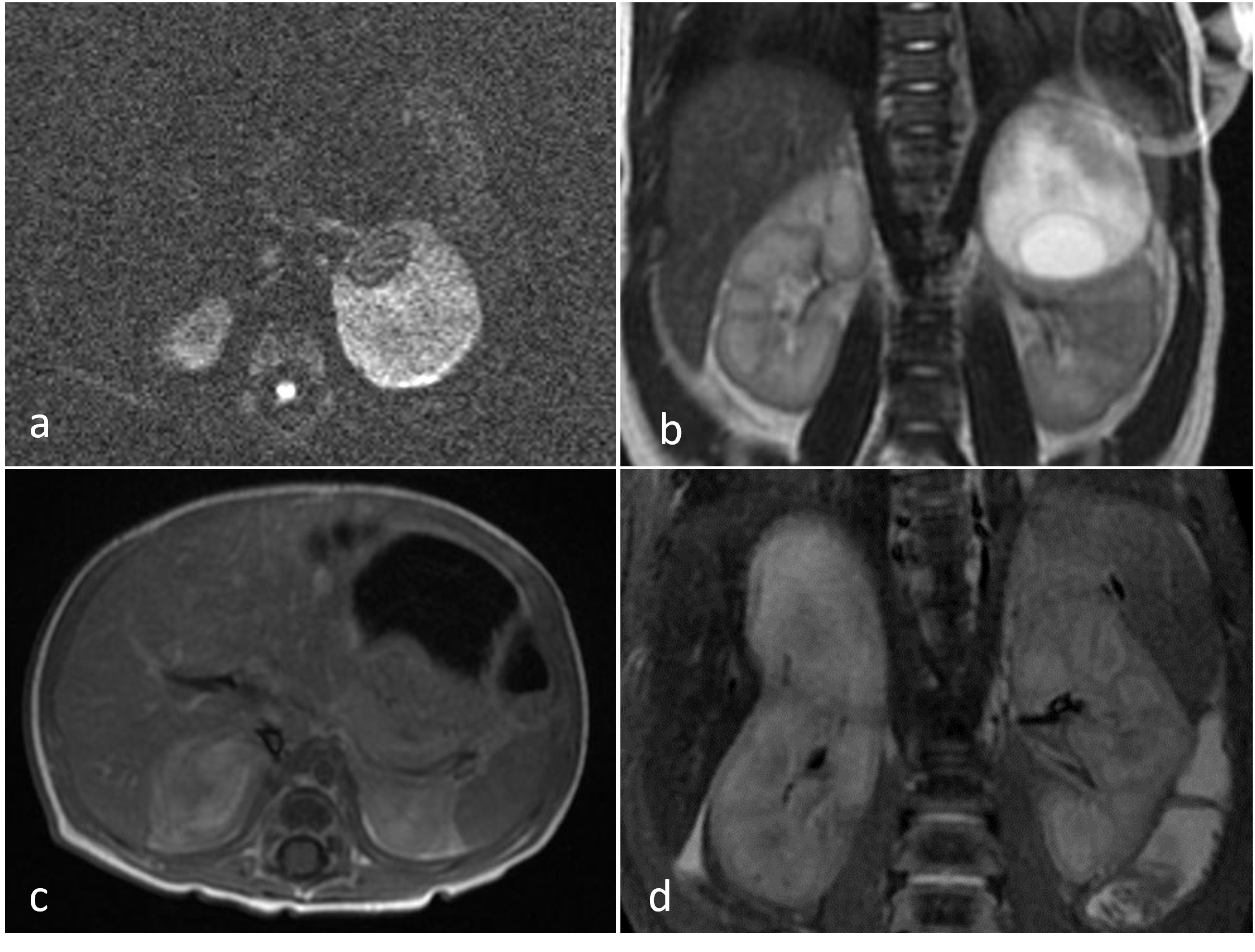

Supplement: Supplementary file 1 [file cancers-16-00043-s001.zip › Supplementary Figure S1.tif]

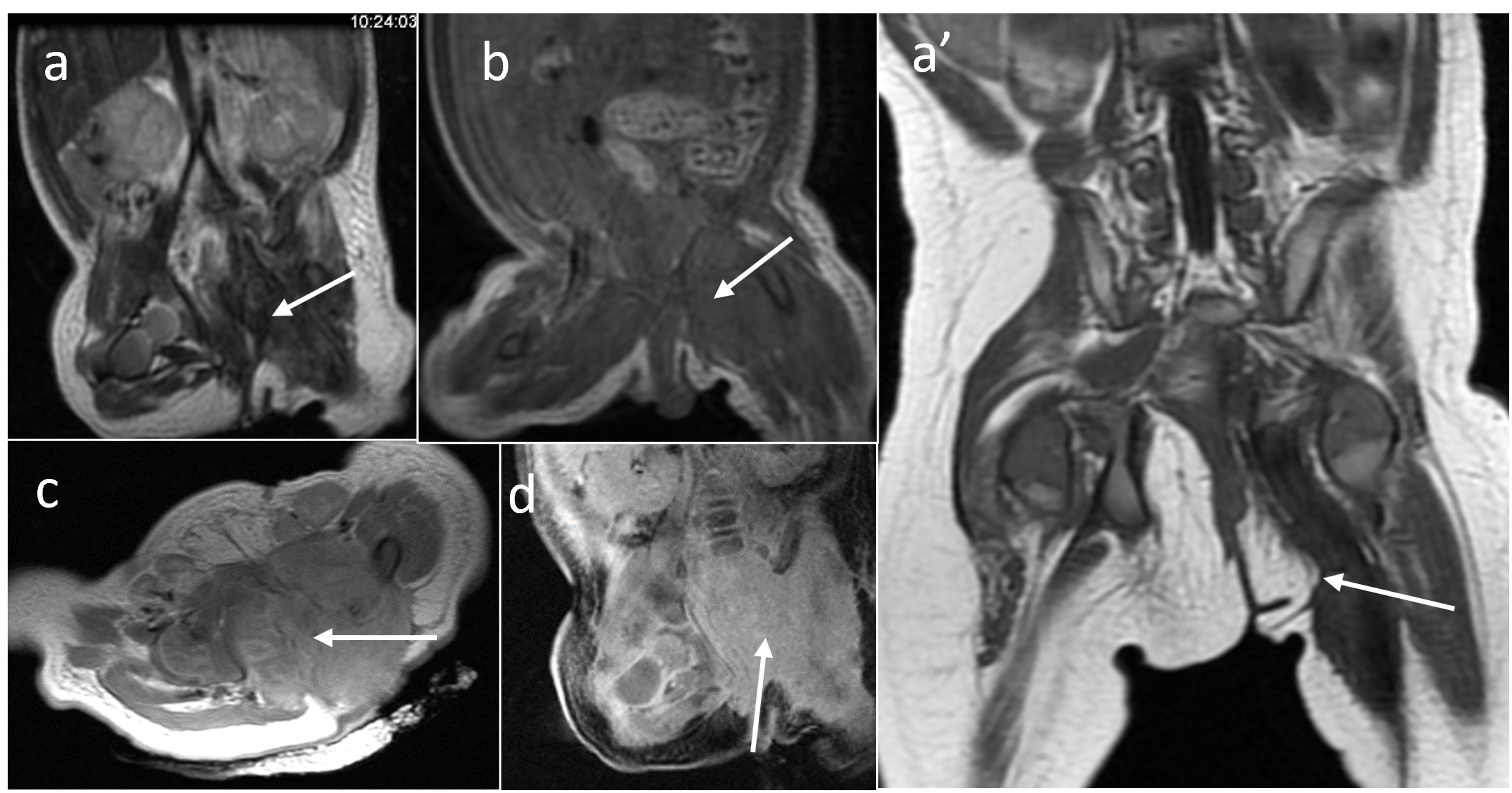

Supplement: Supplementary file 1 [file cancers-16-00043-s001.zip › Supplementary Figure S10.tif]

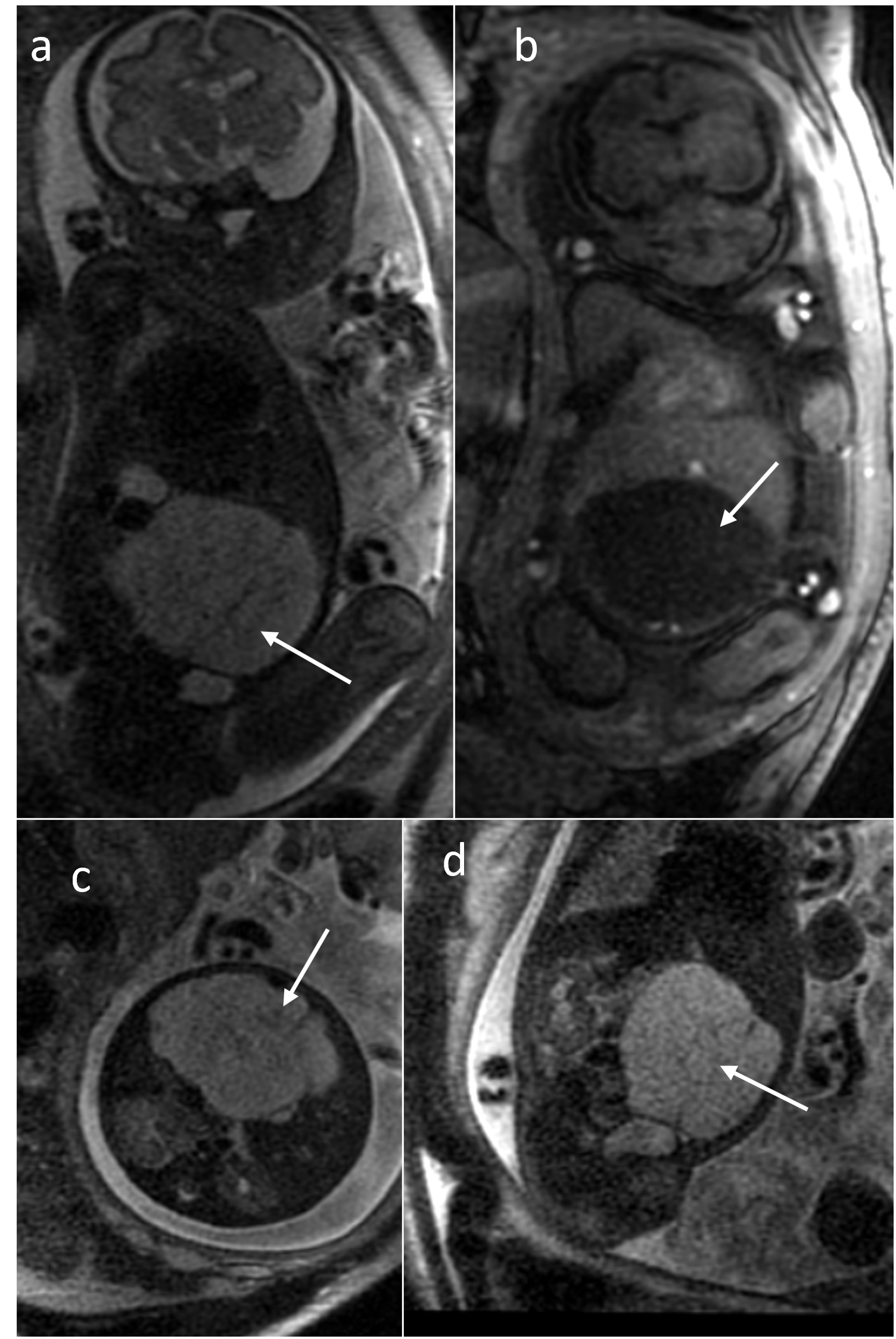

Supplement: Supplementary file 1 [file cancers-16-00043-s001.zip › Supplementary Figure S11.tif]

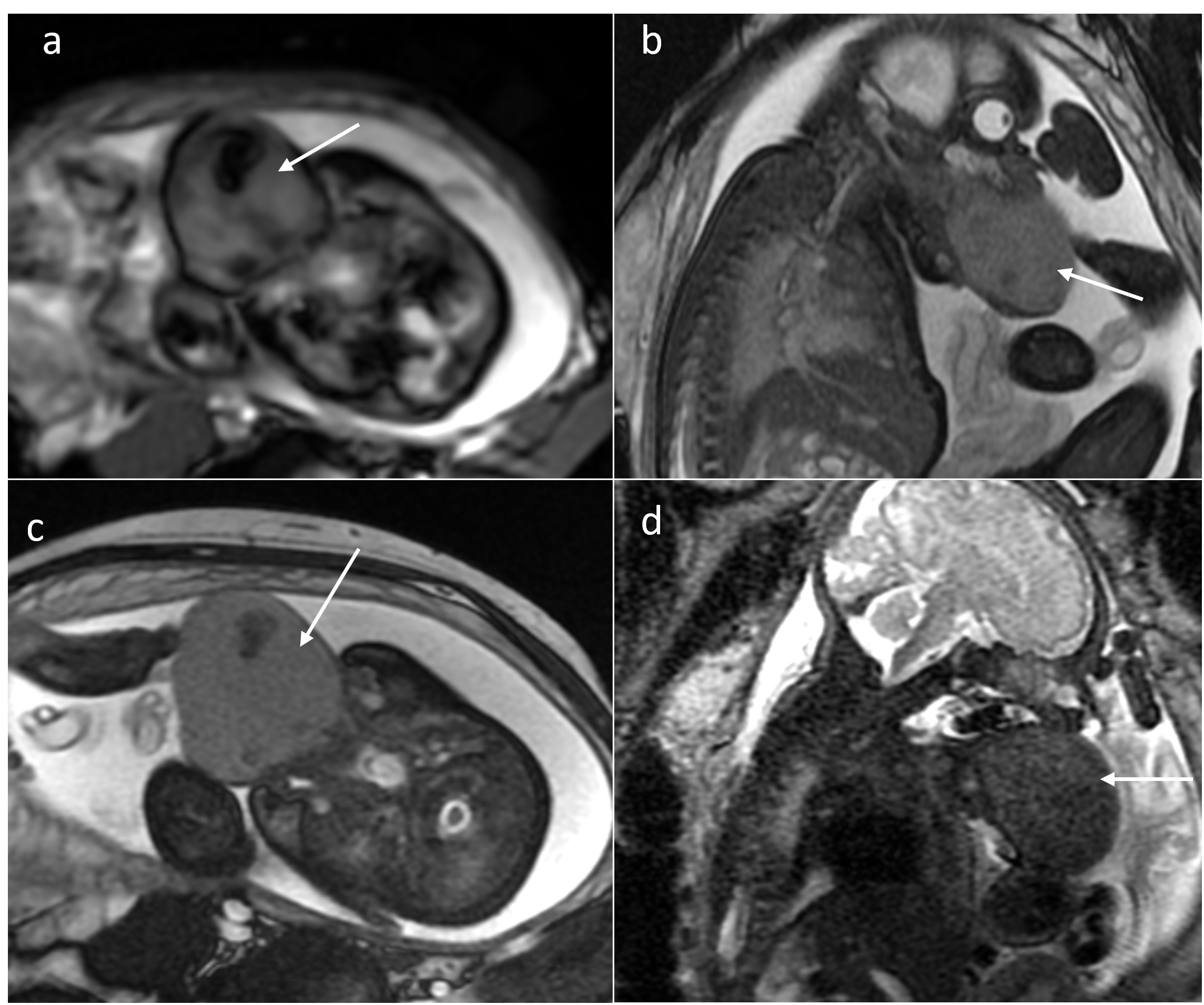

Supplement: Supplementary file 1 [file cancers-16-00043-s001.zip › Supplementary Figure S12.tif]

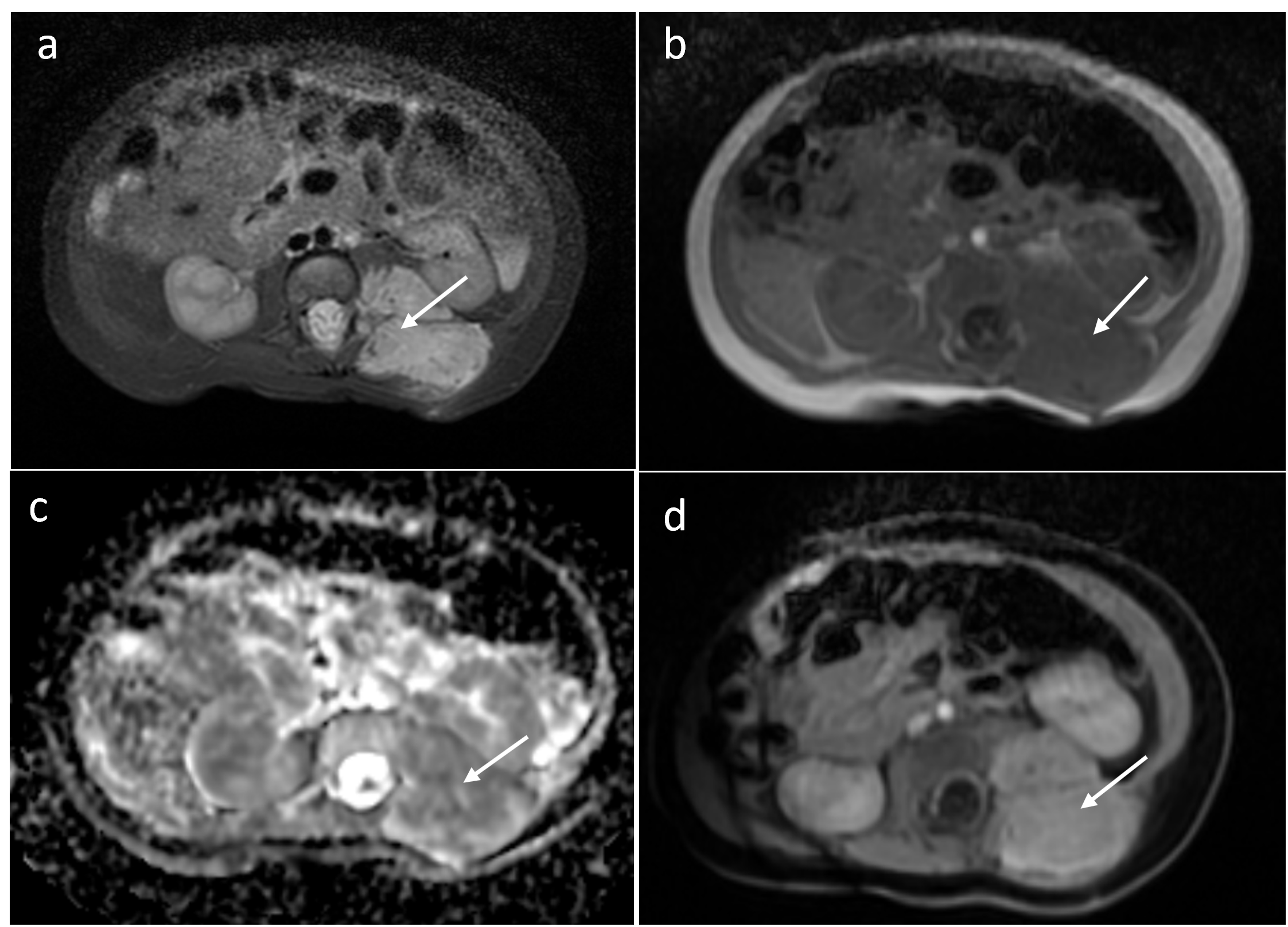

Supplement: Supplementary file 1 [file cancers-16-00043-s001.zip › Supplementary Figure S2.tif]

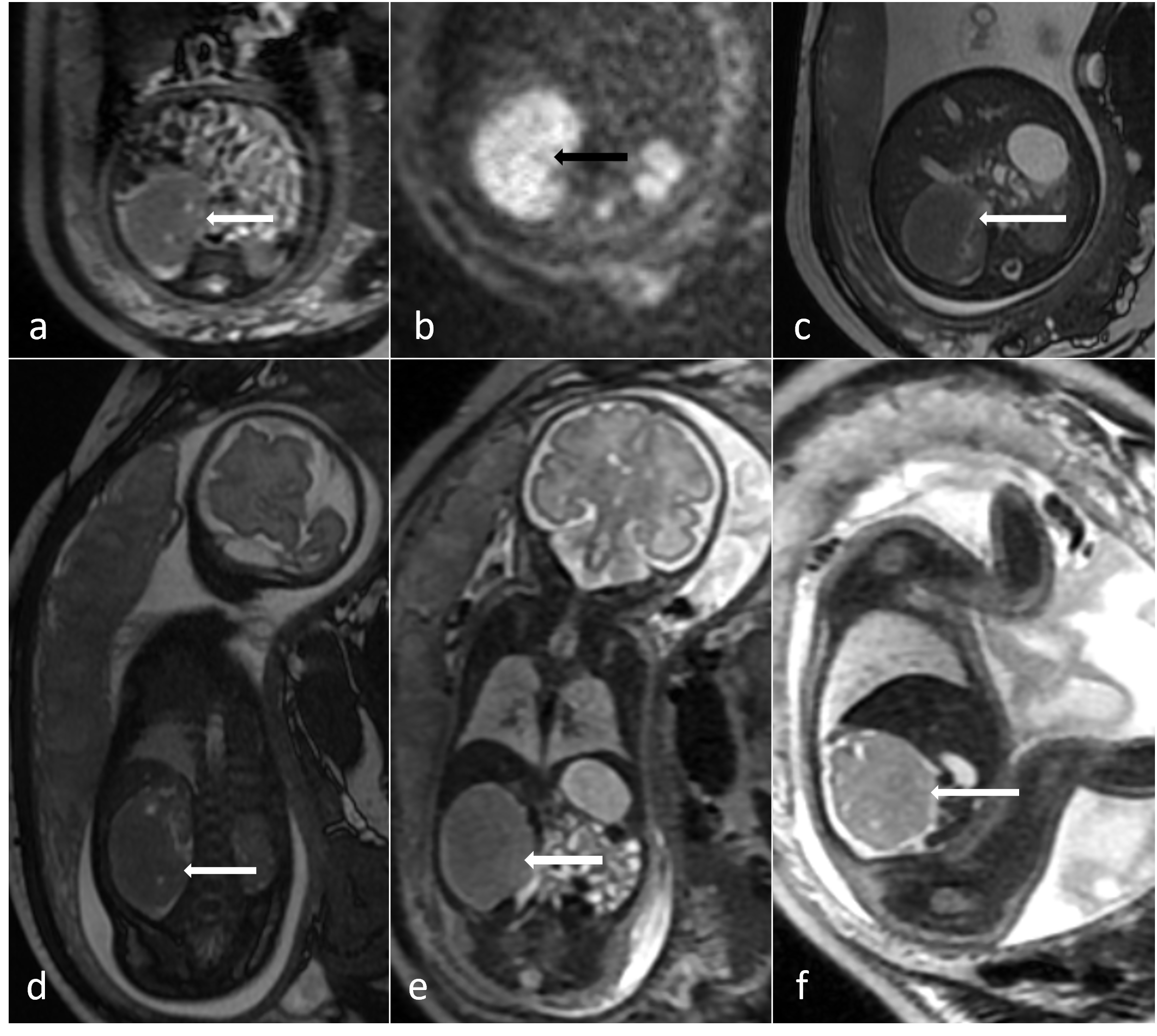

Supplement: Supplementary file 1 [file cancers-16-00043-s001.zip › Supplementary Figure S3.tif]

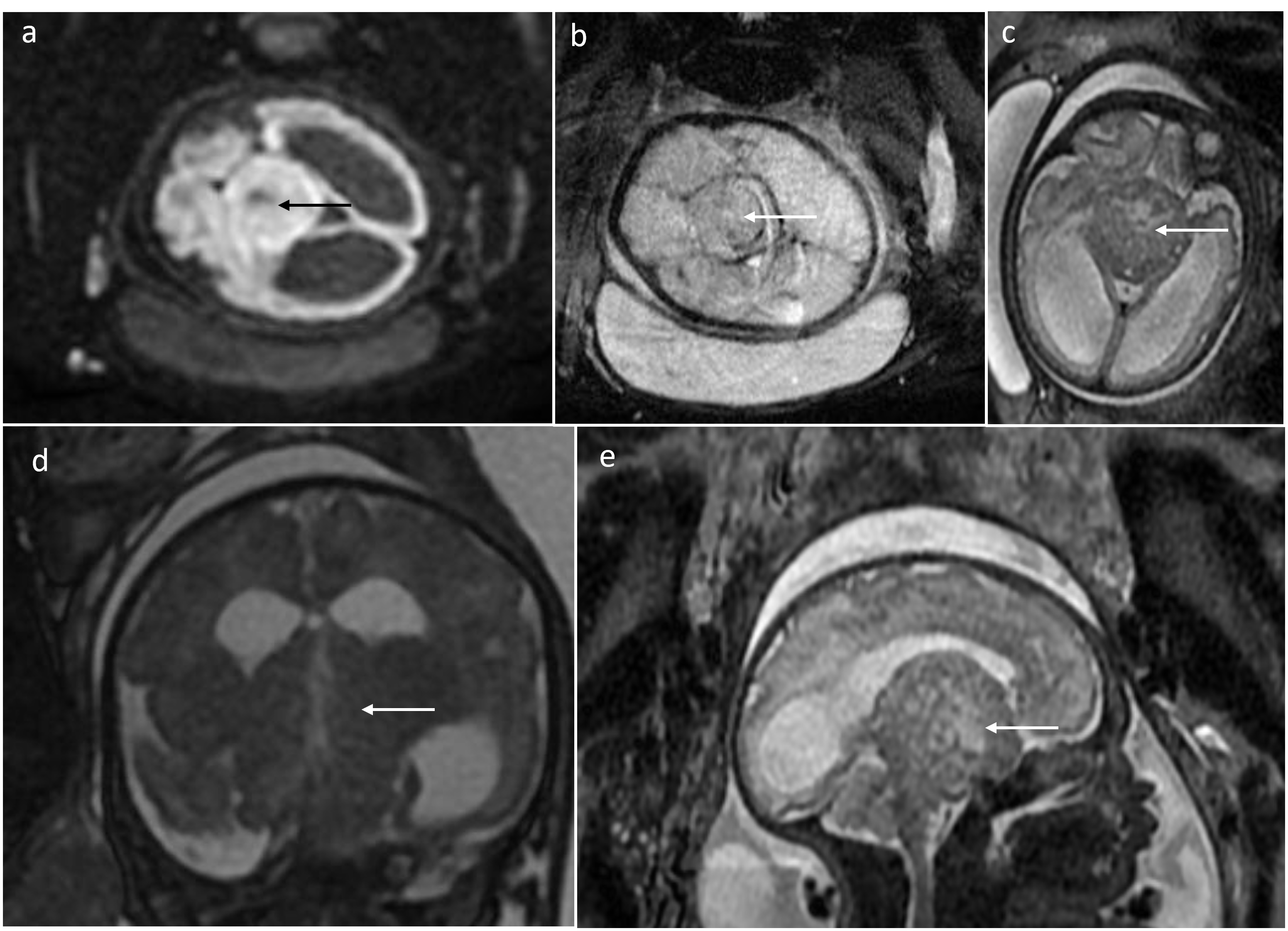

Supplement: Supplementary file 1 [file cancers-16-00043-s001.zip › Supplementary Figure S4.tif]

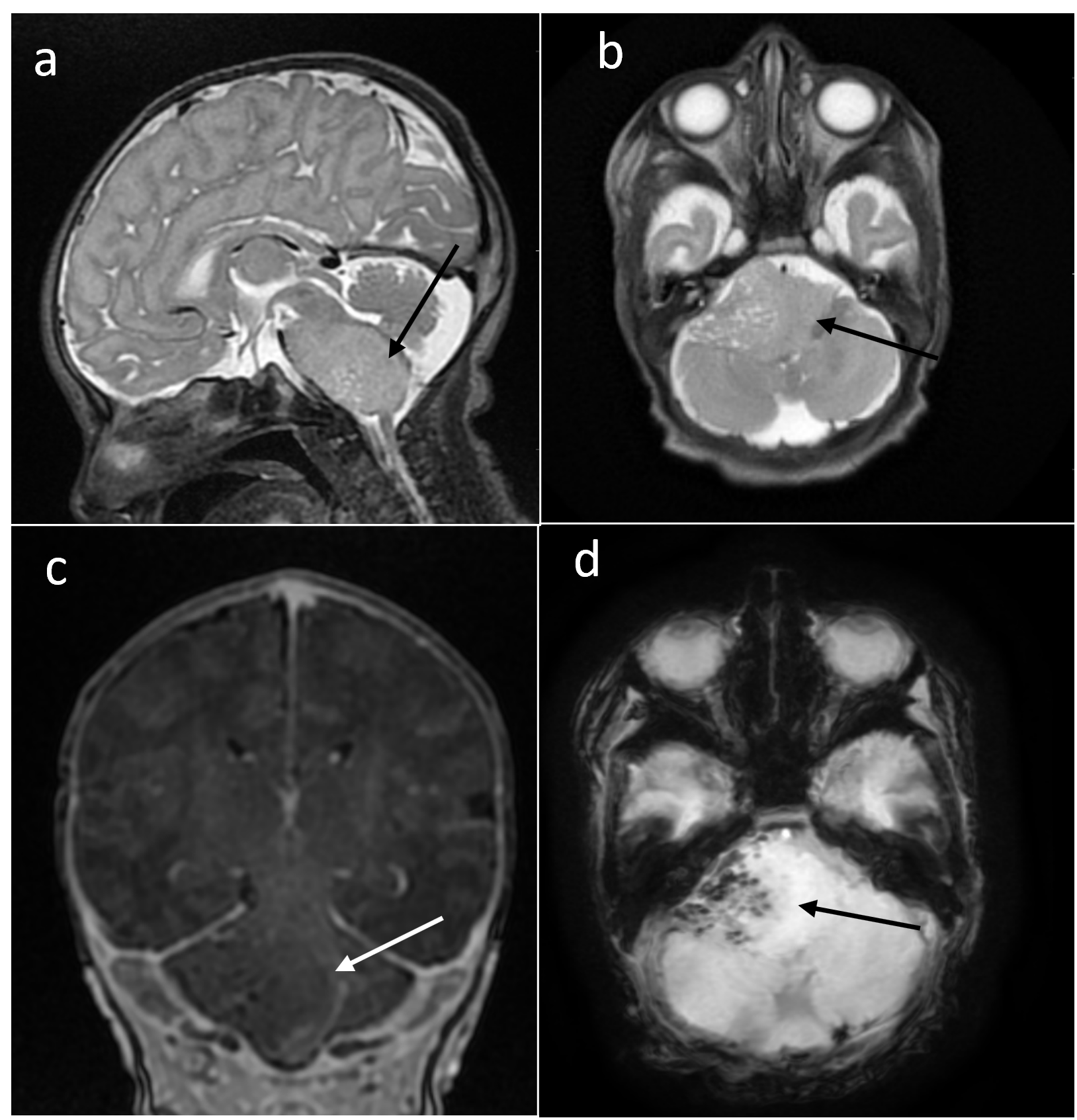

Supplement: Supplementary file 1 [file cancers-16-00043-s001.zip › Supplementary Figure S5.tif]

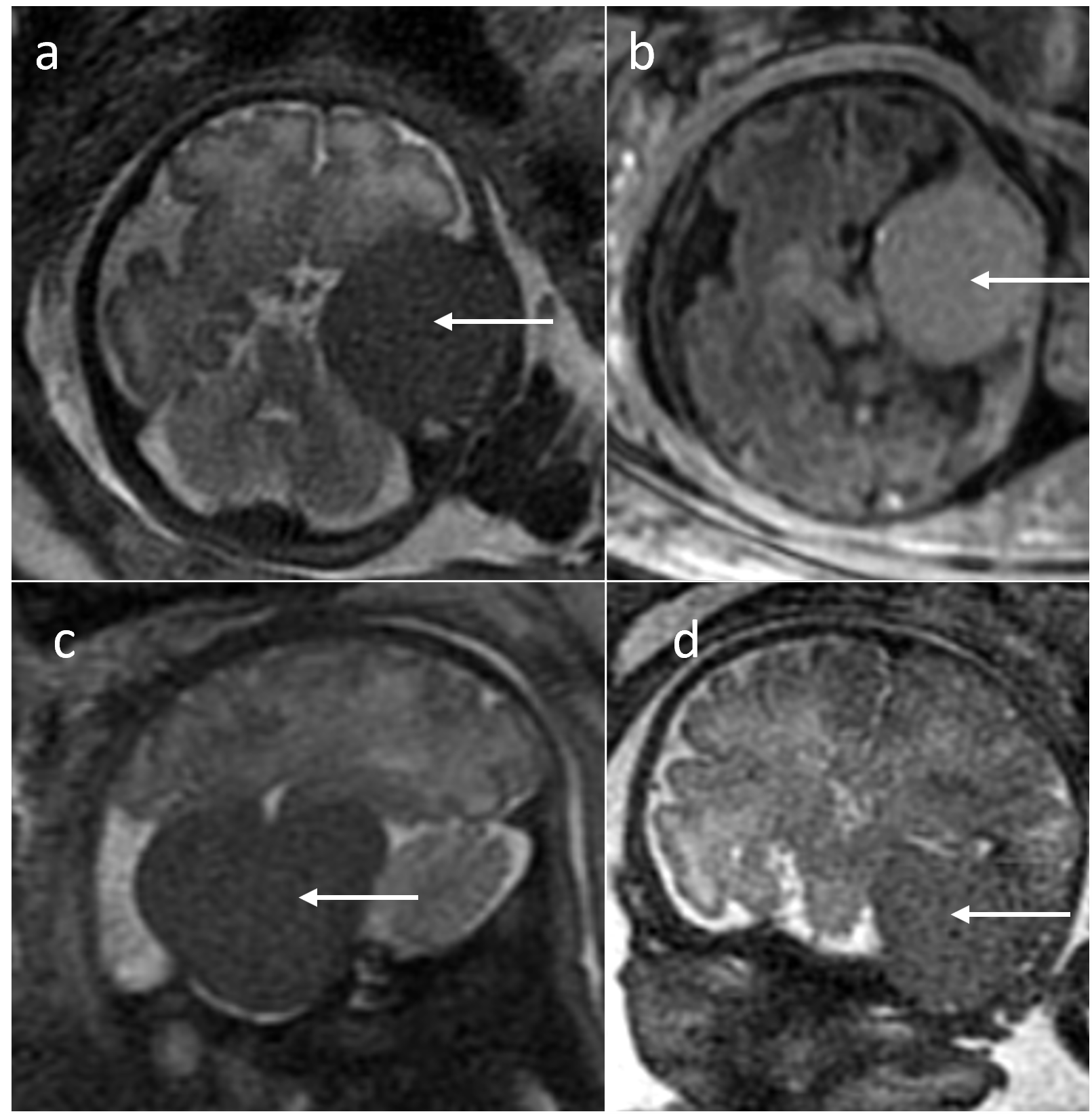

Supplement: Supplementary file 1 [file cancers-16-00043-s001.zip › Supplementary Figure S6.tif]

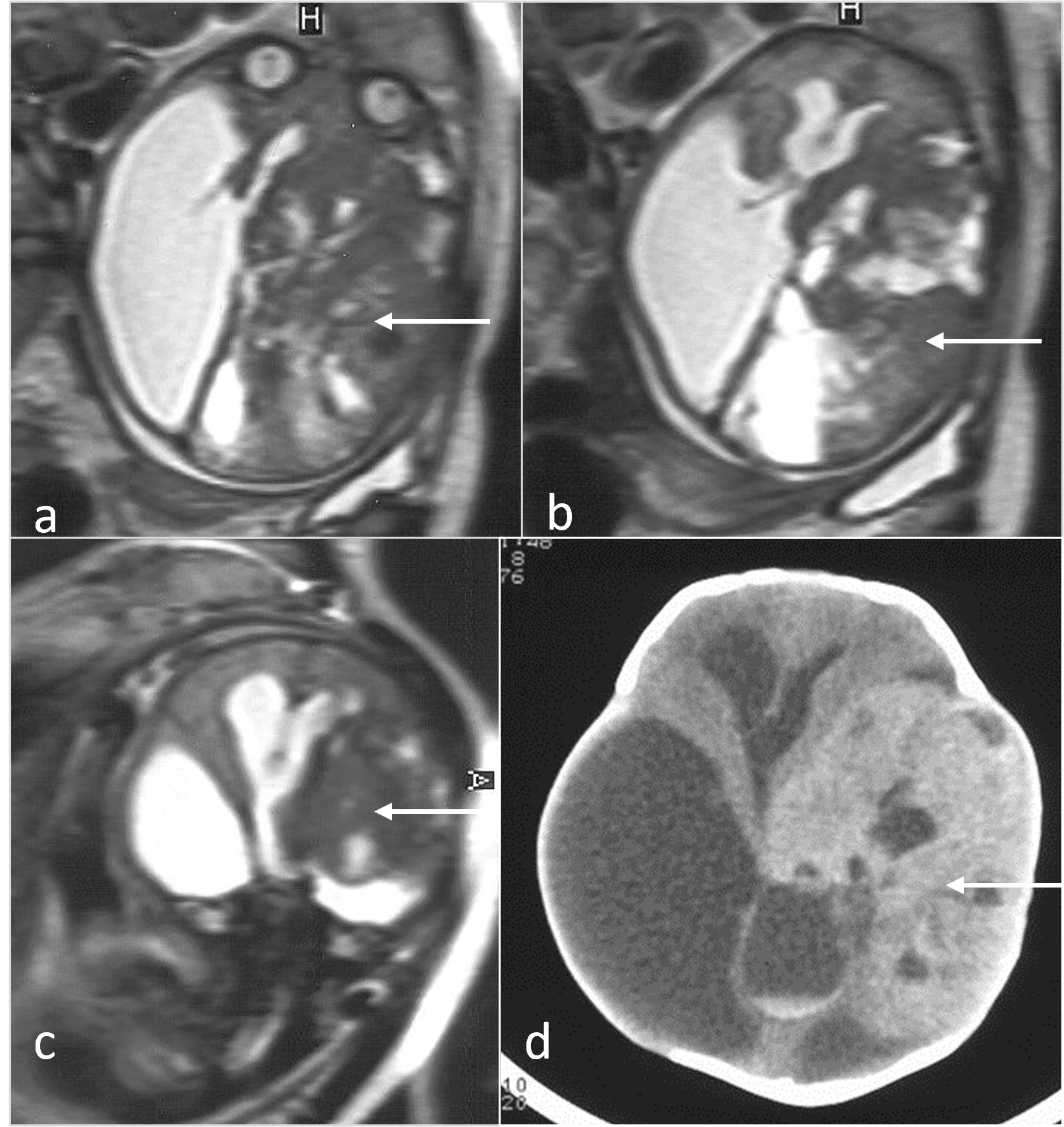

Supplement: Supplementary file 1 [file cancers-16-00043-s001.zip › Supplementary Figure S7.tif]

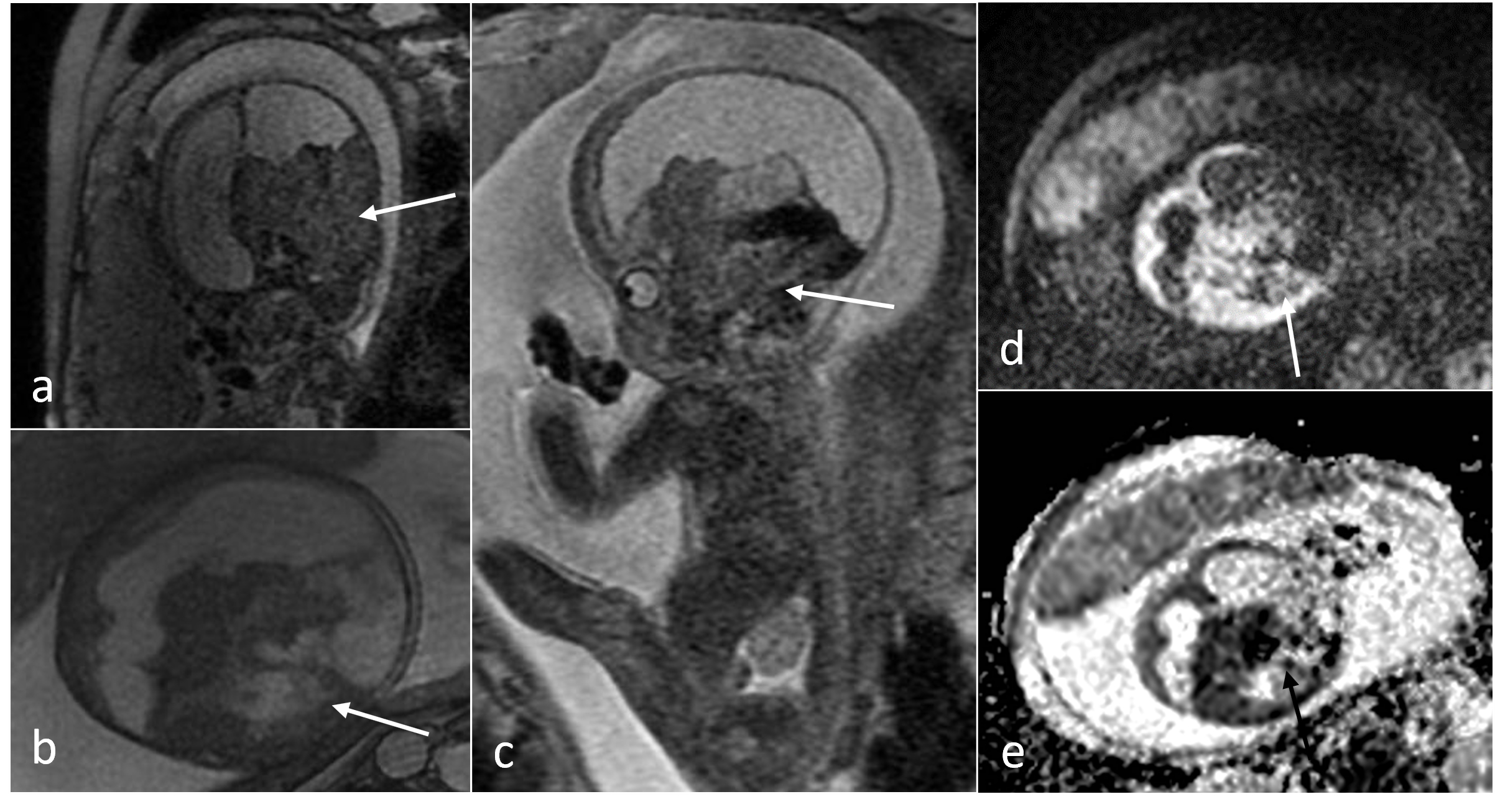

Supplement: Supplementary file 1 [file cancers-16-00043-s001.zip › Supplementary Figure S8.tif]

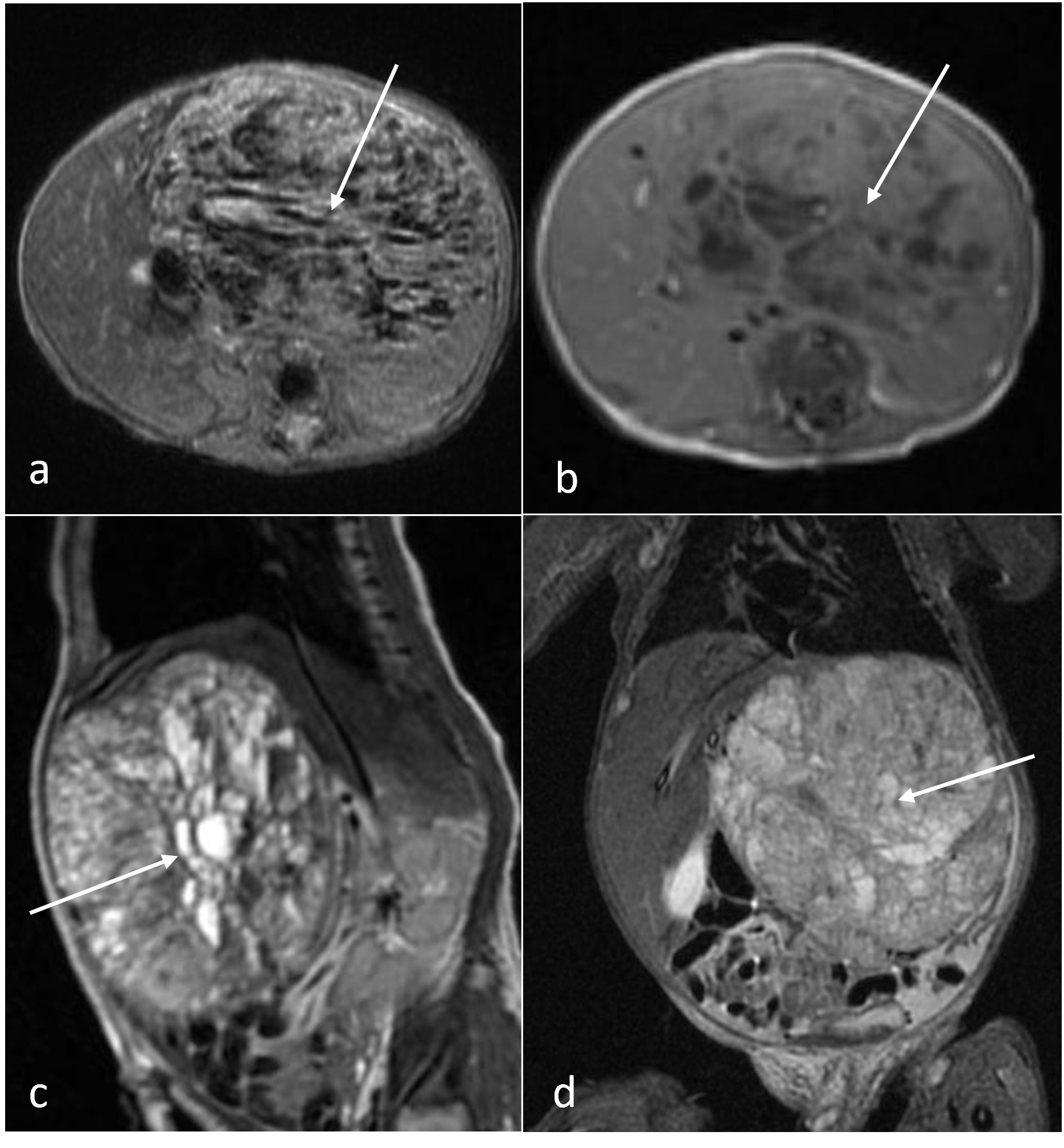

Supplement: Supplementary file 1 [file cancers-16-00043-s001.zip › Supplementary Figure S9.tif]
